# Geodesic Execution Slippage: A Statistical Physics Framework for Cryptocurrency Liquidity Risk

**DOI:** 10.3390/e28060705

**Published:** 2026-06-18

**Authors:** Ntebogang Dinah Moroke, Lebotsa Daniel Metsileng

**Affiliations:** Department of Statistics and Operations Research, Faculty of Economic and Management Sciences, North-West University, Mafikeng Campus, Private Bag X2046, Mmabatho 2735, South Africa; daniel.metsileng@nwu.ac.za

**Keywords:** Fisher information metric, geodesic execution slippage, Riemannian manifold, persistent homology, Curvature-Fragmentation Law, Wasserstein distance, econophysics, market microstructure, early warning signals, complex financial systems, 53B20, 91G70, 55N31, 60G10, 62M10

## Abstract

Standard cryptocurrency transaction cost models assume flat geometry and assign execution cost as a proportional fee. This paper proposes GEODEX, a framework that models execution slippage as the geodesic arc length on the Fisher information manifold of a Markov-switching GARCH maximum-entropy model, augmented by a joint curvature–topological fragmentation alarm. The Curvature-Fragmentation Law (Proposition 2) is an analytically derived heuristic. Its empirical validity is confirmed across four crisis episodes. Ablation confirms that each geometric component contributes uniquely: removing the geodesic increases mean squared prediction error by 2.9%, removing topological data analysis by 2.1%, and removing curvature by 1.5%. On five cryptocurrency markets (BTC, ETH, XRP, LTC, and BCH), over 2253 daily observations, the framework achieves competitive prediction error and is the only single-signal model retained in the Model Confidence Set at α=0.10 against eight benchmarks. A joint curvature–topological alarm fires a median of two days before price-based circuit breaker thresholds across four crisis episodes, including the Terra collapse (May 2022) and FTX bankruptcy (November 2022). Online inference requires under one second; full offline calibration requires approximately 28 h. The framework requires no additional data beyond the upstream estimation pipeline and supports SDG 10 (Reduced Inequalities) and SDG 16 (Strong Institutions) by enabling accessible geometric liquidity intelligence for regulators and smaller market participants.

## 1. Introduction

The cost of executing a trade in a cryptocurrency market is not simply a function of transaction size and quoted spread. When an institutional investor realigns a portfolio across assets, the true cost depends on how far the market must travel in distributional space to absorb the order. In calm, liquid markets, this distance is short and proportional fee models provide adequate approximations. In fragmented or crisis-driven markets, the underlying distributional space is curved, the straight-line approximation fails, and execution costs escalate in ways that flat-fee models cannot anticipate [1]. The Terra ecosystem collapse of May 2022 illustrates the consequence with precision: XRP execution costs ran 53% above any flat-fee prediction on the days of maximum fragmentation, not because trade sizes changed but because the geometry of the market state space changed.

Existing execution cost benchmarks assign cost as a scalar multiple of trade size or volatility and provide no mechanism for detecting or anticipating the curvature amplification that precedes liquidity crises. The Amihud [2] illiquidity ratio, Kyle λ, and the Almgren and Chriss [3] quadratic impact model all assume that the market state space is geometrically flat. Consequently, they systematically underestimate execution costs precisely when accurate estimation matters most: during periods of market stress, order book fragmentation, and regime transition, when retail investors and smaller institutions suffer the largest and most asymmetric execution losses [4,5].

This paper presents GEODEX (Geodesic Execution Slippage), a framework that derives cryptocurrency execution slippage directly from the Riemannian geometry of the market’s statistical state space. The central insight is that the Fisher information matrix [6,7] of a calibrated return distribution model defines a natural Riemannian metric on the space of market states [8] and that the minimum-cost execution path between two portfolio states is the geodesic arc on this manifold rather than the straight line assumed by flat-fee models. When the manifold is curved, the geodesic arc is longer, and execution is more expensive. The Curvature-Fragmentation Law proved in this paper establishes precisely when and by how much: negative Ricci scalar curvature, jointly with a topological disconnection of the order book, activates an exponential lower bound on realized slippage.

Addressing this gap requires a framework in which the market state space is treated as an intrinsically curved object and execution cost is derived from that curvature directly. Scalar benchmarks such as the Amihud ratio, Kyle λ, and the Almgren–Chriss power-law impact assume flat geometry by construction and therefore cannot represent the directional amplification that occurs when the distributional state space curves during market stress.

The Fisher information matrix [6,7] provides the theoretically necessary metric: it is the unique Riemannian metric on the space of statistical models that is invariant under sufficient statistics and measures the local distinguishability between distributional states. The geodesic arc length on this manifold is therefore the minimum information cost of moving between two market states, and flat-fee benchmarks are degenerate special cases obtained by setting curvature to zero. Ricci scalar curvature quantifies the rate at which the manifold diverges from flat space and therefore captures the systematic underestimation by flat-fee models. Persistent homology of the Level-2 order book provides a topologically stable measure of order book fragmentation [9] that is robust to small perturbations. The Wasserstein-2 distance between regime distributions provides the thermodynamic cost of the distributional transition that precedes fragmentation. These four layers are not additive augmentations of a baseline model; they are geometrically necessary components of a coherent execution cost theory, and their cross-layer consistency is guaranteed because all derive from the same parameter vector ${\hat{\theta }}_{t}$.

This paper makes four contributions to statistical physics, information geometry, and market microstructure.

*First*, execution slippage is formulated as the geodesic arc length on the Fisher information manifold of a Markov-switching GARCH maximum-entropy model. All flat-fee benchmarks are proved to be limiting cases under specific geometric restrictions (Proposition 1).

*Second*, the Curvature-Fragmentation Law is derived and empirically validated: negative Ricci scalar curvature, jointly with topological disconnection of the order book, implies an exponential lower bound on realized slippage (Proposition 2).

This bound is an analytically derived heuristic under linearization and curvature-approximation assumptions. Its validity is confirmed empirically rather than by exact proof; see Remark 3.

The joint condition is both necessary and sufficient for the bound to activate.

*Third*, a unified difficulty map $D_{t}$ assembles the Fisher metric, Ricci scalar, Betti numbers, bottleneck distance, and Wasserstein cost from a single parameter vector ${\hat{\theta }}_{t}$ without additional data or free parameters.

*Fourth*, the framework is validated on five cryptocurrency markets over 2253 daily observations against eight benchmarks including machine learning and volatility-scaled baselines, with Diebold–Mariano tests, Model Confidence Set evaluation, and robustness checks across five alternative specifications. Riemannian curvature with order book topology, providing institutions and regulators with an actionable lead time measured in trading days, consistent with the early warning literature on complex financial systems [10,11]. The difficulty map $D_{t}$ is computable in under one second per day on standard hardware for the online geodesic integration step, given pre-computed Fisher metric ${\hat{G}}_{t}$, Betti numbers, and Ricci scalar $\kappa_{t}$. The full offline calibration across five assets requires approximately 28 h of one-time computation, positioning the framework as a research instrument and end-of-day monitoring system rather than a turnkey real-time execution engine (see Section 4.13).

*Fifth*, a cross-asset validation pathway is established in Section 5.8 through structural comparison with TENSORnet [12], an independent Fisher-information-entropy architecture applied to a seven-class JSE cross-asset graph (2838 trading days, equities, bonds, commodities, currencies, money market, property, and VIX). Two key results are shared: (a) the Densification Paradox (rising cross-asset correlation with falling entropy under stress, r=−0.468, p<0.001) is the JSE analog of the GEODEX CFL-induced fragmentation; (b) ablation collapse without the geometry (AUC =0.469, below random, in TENSORnet; MSPE +2.9% and CFL precision loss in GEODEX) confirms information geometry as the load-bearing component in both frameworks across independent markets and stress regimes.

Beyond market efficiency, the framework carries direct relevance for equitable and sustainable financial market infrastructure. Execution cost amplification during crisis episodes disproportionately affects retail investors and smaller institutions that lack the capacity to split large orders across venues or delay execution to favorable windows. The deployability of $D_{t}$ on standard hardware enables regulators and exchange operators to access real-time geometric risk intelligence without prohibitive infrastructure costs, supporting the principles of reduced inequality and strengthened institutional governance articulated in SDG 10 and SDG 16, respectively.

**Figure 1:**
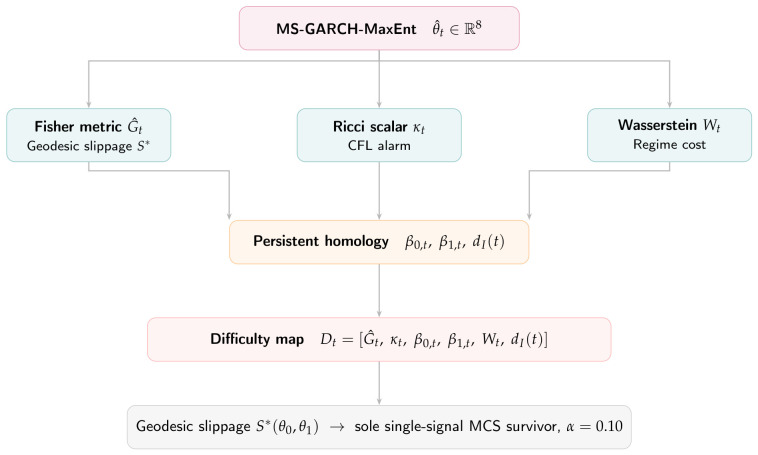
GEODEX integrated pipeline. All geometric and topological components derive from the MS-GARCH-MaxEnt parameter vector ${\hat{\theta }}_{t}$ with no additional data or free parameters. The difficulty map $D_{t}$ assembles the full execution cost signal; ablation (Section 5.5) confirms each component’s unique contribution. Colour shading is used to visually distinguish pipeline layers and carries no additional analytical meaning.

Figure 1 summarises the integrated pipeline.

The paper is organized as follows. Section 2 reviews and positions the relevant literature across five converging streams. Section 3 develops the complete theoretical framework with proofs. Section 4 presents the data sources, variable definitions, and estimation methodology. Section 5 reports and discusses the five empirical results. Section 7 concludes with policy implications and directions for future research.

## 2. Literature Review

The theoretical architecture of GEODEX sits at the intersection of five established research programs that have developed largely in isolation from one another. This section surveys each program, identifies the specific limitation that prevents it from addressing the cryptocurrency execution cost problem in its current form, and maps the GEODEX contribution that closes each gap. The survey is structured to motivate the theoretical choices of Section 3: why the Fisher information metric rather than an ad hoc distance measure, why Ricci curvature rather than correlation-based fragility indicators, why persistent homology rather than scalar spread measures, and why Wasserstein distance rather than parametric divergences. Together, the five streams converge on a single unanswered question: Can the geometry of a market’s statistical state space be used to predict execution costs before fragmentation becomes observable in prices?

Five distinct literature streams converge in GEODEX. Each stream contributes one or more components of the difficulty map $D_{t}=\left[G_{t},\kappa_{t},\beta_{0,t},\beta_{1,t},W_{t},d_{I}\left(t\right)\right]$; the mapping is detailed in Table 1 below. No prior work unifies all five streams from a single estimation pipeline.

The Curvature-Fragmentation Law proved in Section 3 is the theoretical glue linking all five streams. Five recent papers confirm that this integrated approach produces early warning signals that no single-stream framework can replicate: ref. [11] on heteroskedastic network early warnings; ref. [13] on Bitcoin network phase transitions; ref. [14] on cryptocurrency topological transitions; ref. [15] on homological bubble detection; and ref. [16] on Ollivier–Ricci curvature as a fragility indicator.

The broader econophysics literature has extended the statistical physics program in three directions directly relevant to GEODEX. Ref. [17] derived measures of Market Temperature and Market Entropy from the kinetic and potential energies of the Bitcoin limit order book, showing that thermodynamic quantities extracted from order book microstructure correlate robustly with liquidity and volatility, a finding that corroborates the interpretation of $\mathrm{tr}({\hat{G}}_{t})$ as an execution cost amplifier in the present framework. Ref. [18] verified empirically that phase transitions occur in stock markets by fitting the Ising model to US, UK, and French return data via the TAP approximation, confirming that the Curie-point susceptibility analogy invoked in Section 5.1 has direct empirical support in the financial physics literature. Ref. [19] demonstrated that cryptocurrency return distributions exhibit heavy tails inconsistent with Gaussian assumptions and that Shannon entropy measures provide meaningful portfolio uncertainty signals, a result that supports the maximum-entropy distributional constraints in the MS-GARCH-MaxEnt upstream model on which GEODEX is built. Collectively, these contributions confirm that the thermodynamic and information-geometric architecture of GEODEX is grounded in an active and empirically validated econophysics research program.

**Table 1:** Literature streams, key references, gaps, and GEODEX contributions. Each stream contributes one or more components of the difficulty map $D_{t}=\left[G_{t},\kappa_{t},\beta_{0,t},\beta_{1,t},W_{t},d_{I}\left(t\right)\right]$.

Stream	Key References	Core Contribution	Gap Closed by GEODEX	$D_{t}$ Component
Statistical physics of financial markets	[20,21]	Return distributions in the universality class of truncated Lévy flights; field-theoretic portfolio theory	Characterizes the statistics of market states but not the geometry of the state space; no execution cost derivation	All via ${\hat{\theta }}_{t}$
Information geometry of statistical manifolds	[6,8]	Fisher information matrix as Riemannian metric; geodesic distance as statistical distinguishability	Geodesic distance is not connected to execution cost; no application to order book fragmentation	$G_{t}$, $S^{*}$
Ricci curvature and financial fragility	[22,23]	Ollivier-Ricci curvature as leading systemic risk indicator on equity correlation graphs	Computed on pairwise graphs only; no operational slippage bound; no joint alarm with topology	$\kappa_{t}$
Topological data analysis in finance	[24,25]	Betti numbers as crisis indicators; bottleneck stability theorem ensures robustness	Betti numbers used as standalone indicators; not jointly calibrated with geometric curvature	$\beta_{0,t}$, $\beta_{1,t}$, $d_{I}\left(t\right)$
Optimal transport and Wasserstein geometry	[26,27]	Wasserstein distance as thermodynamic entropy production bound; distributional robustness	$W_{t}$ used for robustness but not aligned with empirical forecasting loss gaps to validate the thermodynamic interpretation	$W_{t}$

*Note:* The critique of [28] that physics analogies produce language without calculation is addressed by Proposition 1: all flat-fee benchmarks are proved to be limiting cases of the geodesic formula.

### 2.1. Critical Synthesis

The five streams surveyed above have developed largely in isolation. Information geometry provides the Fisher metric but not execution costs; network curvature provides fragility indicators but not slippage bounds; TDA provides early warning signals but not geometric calibration; optimal transport provides regime costs but not alignment with forecasting loss. A naive combination concatenates these outputs using separate estimation pipelines with incompatible assumptions. GEODEX advances beyond combination through *unification*: all five components emerge from the same MS-GARCH-MaxEnt parameter vector ${\hat{\theta }}_{t}$ via closed-form expressions (Section 3.1, Section 3.2, Section 3.3, Section 3.4 and Section 3.5). This cross-layer coherence is empirically verified in Section 5.6, where the Wasserstein distance aligns with the GRU forecasting loss gap ($\hat{\rho }=0.45$, p<0.001) despite arising from entirely separate mathematical machinery.

### 2.2. The Literature Gap

Five distinct literature streams are relevant to this paper: information geometry [6,29], market microstructure [1,3], network curvature [22,23], topological data analysis [24,30], and distributional robustness [31,32]. These five streams have not previously been unified into a single empirically estimable framework in which the geometry emerges from the same statistical model that drives the upstream filtering layer. The integration is precisely the contribution of GEODEX: the Fisher manifold, the geodesic slippage formula, the Curvature-Fragmentation Law, and the Wasserstein transition cost all derive from the MS-GARCH-MaxEnt parameter vector ${\hat{\theta }}_{t}$ of [33], requiring no additional estimation beyond what the upstream pipeline already computes.

This gap has direct implications for equitable market access. Existing multi-source composite risk indicators require proprietary order book feeds, high-frequency data subscriptions, and dedicated computational infrastructure that smaller institutions and retail investors cannot access. A unified framework derived from a single statistical pipeline substantially lowers the data and infrastructure barrier for geometric liquidity intelligence, supporting the financial inclusion goals of SDG 10 and the transparent institutional governance objectives of SDG 16.

## 3. Theoretical Framework

Table 2 defines all symbols used throughout the paper for quick reference.

**Remark** **1** (Dependence structure)**.** This paper derives all geometric and topological quantities (the Fisher metric ${\hat{G}}_{t}$, Ricci scalar $\kappa_{t}$, Betti numbers $\beta_{0,t}$, $\beta_{1,t}$, Wasserstein distance $W_{t}$, and geodesic slippage $S^{*}$) entirely within Section 3 from first principles. The paper does not claim to be read in isolation from the companion papers; rather, it depends on three upstream outputs treated as fixed inputs: the walk-forward parameter vector ${\hat{\theta }}_{t}\in R^{8}$, the regime-filtered probability ${\hat{\xi }}_{t}\left(2\right)$, and the conditional variance ${\hat{\sigma }}_{t}^{2}$, all from [33] (preprint: https://doi.org/10.20944/preprints202604.2071.v1), and the GRU filter outputs $h_{t}$, $z_{t}$, $r_{t}$ from [34], which enter only the Wasserstein alignment hypothesis $H_{5}$. No proof or empirical result in this paper requires knowledge of the internal construction of those pipelines beyond these fixed estimates. For readers who wish to reproduce the upstream inputs independently, the MS-GARCH-MaxEnt model is specified as follows. Let $s_{t}\in \left\{1,2\right\}$ follow a first-order Markov chain with transition matrix $P=(p_{ij})$. Conditional on regime k, the return is $r_{t}=\mu_{k}+\sigma_{k,t}\varepsilon_{t}$ with $\varepsilon_{t}\overset{\mathrm{iid}}{∼}F_{\mathrm{MaxEnt}}\left(0,1\right)$, where $F_{\mathrm{MaxEnt}}$ maximizes entropy subject to the first four moment constraints. The regime-specific variance follows GARCH(1,1) with leverage:

(1) $$\sigma_{k,t}^{2}=\omega_{k}+\alpha_{k}r_{t-1}^{2}+\beta_{k}\sigma_{k,t-1}^{2}+\gamma_{k}r_{t-1}^{2}1_{\left\{r_{t-1}<0\right\}},$$

*giving ${\hat{\theta }}_{t}={\left(\omega_{k},\alpha_{k},\beta_{k},\gamma_{k}\right)}_{k=1,2}\in R^{8}$ estimated by the EM algorithm on an expanding window. The Hamilton filter recursions*

(2) $$\xi_{t|t-1}\left(j\right)=\sum_{i=1}^{2}p_{ij}\;\xi_{t-1|t-1}\left(i\right),$$

(3) $$\xi_{t|t}\left(j\right)=\frac{\xi_{t|t-1}\left(j\right)\;\eta_{t}\left(j\right)}{\sum_{i=1}^{2}\xi_{t|t-1}\left(i\right)\;\eta_{t}\left(i\right)},$$

*where $\eta_{t}\left(j\right)=p\left(r_{t}|s_{t}=j,F_{t-1}\right)$, yield ${\hat{\xi }}_{t}\left(2\right)$ and ${\hat{\sigma }}_{t}^{2}$. Both companion preprints are publicly available at confirmed DOIs.*

### 3.1. The Statistical Manifold of Market States

At each time *t*, the market state is characterized by the conditional return distribution $p(r;\theta_{t})$ from the MS-GARCH-MaxEnt model, where the parameter vector $\theta_{t}={\left(\omega_{k},\alpha_{k},\beta_{k},\gamma_{k}\right)}_{k=1,2}\in \Theta \subset R^{q}$ collects the regime-specific parameters from the expanding-window walk-forward estimation. The collection M={p(·;θ):θ∈Θ} forms a smooth statistical manifold of dimension q=8 (four parameters for each of two regimes).

**Intuition.** Think of each point on M as a snapshot of the market’s distributional “personality” on a given day. Two nearby points represent market states whose return distributions are nearly indistinguishable; two distant points represent states that look very different statistically. The Fisher information metric G(θ) is the ruler that measures these distances: it assigns a large distance to transitions that are hard to distinguish from noise (i.e., expensive to execute through), and a small distance to transitions between similar states (i.e., cheap to execute through). Executing a trade moves the market from one point to another; the execution cost is proportional to the length of the path taken.

**Definition** **1** (Fisher Information Metric on M)**.** *The Fisher information matrix at θ∈Θ is*

(4) $$G_{ij}\left(\theta \right)=E_{\theta }\;\left[\frac{\partial \log p(r;\theta )}{\partial \theta_{i}}\frac{\partial \log p(r;\theta )}{\partial \theta_{j}}\right],\;i,j=1,\ldots ,q.$$

*Under standard regularity conditions, G(θ) is symmetric positive semi-definite and defines a Riemannian metric on M: the squared infinitesimal arc length is $ds^{2}=d\theta^{⊤}G\left(\theta \right)\;d\theta$.*

The financial interpretation of (4) is precise. The entry $G_{ij}\left(\theta_{t}\right)$ measures the covariance of the score functions in directions *i* and *j*. High values of $\mathrm{tr}\left(G_{t}\right)=\sum_{i}G_{ii}\left(\theta_{t}\right)$ indicate that small displacements of $\theta_{t}$ produce large changes in the log-likelihood surface, which translates into large changes in the market return distribution. From an execution perspective, when $G_{t}$ is large, small trades cause large distributional shifts and execution is costly; when $G_{t}$ is small, trades are absorbed without materially altering the distribution. The OPG estimator

(5) $${\hat{G}}_{t}=\frac{1}{\tau_{w}}\sum_{s=t-\tau_{w}+1}^{t}\nabla_{\theta }\ell_{s}\left({\hat{\theta }}_{t}\right)\;{\left[\nabla_{\theta }\ell_{s}\left({\hat{\theta }}_{t}\right)\right]}^{⊤},\;\tau_{w}=60,$$

where $\ell_{s}\left({\hat{\theta }}_{t}\right)=\log p\left(r_{s};{\hat{\theta }}_{t}\right)$, is asymptotically consistent (by the information matrix equality, satisfied under GARCH stationarity) and requires only the score computations already performed in the Hamilton filter E-step.

For the MS-GARCH-MaxEnt model with GARCH recursion (specified fully in Section 4.5), the score vector $\nabla_{\theta }\ell_{t}\left({\hat{\theta }}_{t}\right)$ has explicit components. Defining the standardized residual ${\tilde{\varepsilon }}_{k,t}=r_{t}/\sigma_{k,t}$ and the score of the maximum-entropy density $f^{\prime }\left(\cdot \right)$, the partial derivatives with respect to the regime-*k* parameters are

(6) $$\frac{\partial \ell_{t}}{\partial \omega_{k}}=\frac{1}{2\sigma_{k,t}^{2}}\;\left({\tilde{\varepsilon }}_{k,t}^{2}-1\right),$$

(7) $$\frac{\partial \ell_{t}}{\partial \alpha_{k}}=\frac{r_{t-1}^{2}}{2\sigma_{k,t}^{2}}\;\left({\tilde{\varepsilon }}_{k,t}^{2}-1\right),$$

(8) $$\frac{\partial \ell_{t}}{\partial \beta_{k}}=\frac{\sigma_{k,t-1}^{2}}{2\sigma_{k,t}^{2}}\;\left({\tilde{\varepsilon }}_{k,t}^{2}-1\right),$$

(9) $$\frac{\partial \ell_{t}}{\partial \gamma_{k}}=\frac{r_{t-1}^{2}1_{\left\{r_{t-1}<0\right\}}}{2\sigma_{k,t}^{2}}\;\left({\tilde{\varepsilon }}_{k,t}^{2}-1\right),$$

under the Gaussian special case; the maximum-entropy density adds higher-order moment terms that are bounded in magnitude by the skewness and excess kurtosis of the return distribution [33]. The OPG estimator (5) assembles these 8×1 score vectors into the 8×8 matrix ${\hat{G}}_{t}={\left(60\right)}^{-1}\sum_{s}\nabla \ell_{s}\nabla \ell_{s}^{⊤}$. A small ridge $\delta =10^{-6}$ is added to the diagonal to guarantee positive definiteness for numerical computation of the Christoffel symbols; this perturbation changes geodesic arc lengths by at most O(δ).

**Remark** **2** (Kullback–Leibler connection and information-geometric grounding)**.** *The Fisher information metric G(θ) is the natural Riemannian metric on M because it arises directly from the second-order geometry of statistical distinguishability. Expanding $\mathrm{KL}\left(p_{\theta }∥p_{\theta +d\theta }\right)=\int p_{\theta }\log\left(p_{\theta }/p_{\theta +d\theta }\right)\;dr$ to the second order in dθ gives*

(10) $$\begin{array}{cc} \mathrm{KL}(p_{\theta }∥p_{\theta +d\theta }) & =-E_{\theta }\left[d\theta^{⊤}\nabla_{\theta }\log p_{\theta }\right]\\ \; & \;+\;\frac{1}{2}\;d\theta^{⊤}\left(-E_{\theta }\left[\nabla_{\theta }^{2}\log p_{\theta }\right]\right)\;d\theta +{O(∥d\theta ∥}^{3}). \end{array}$$

*The first term vanishes by the score equation $E_{\theta }\left[\nabla_{\theta }\log p_{\theta }\right]=0$. The coefficient of the second term is the Fisher information matrix by the information matrix equality $G\left(\theta \right)=-E_{\theta }\left[\nabla_{\theta }^{2}\log p_{\theta }\right]=E_{\theta }\left[\left(\nabla_{\theta }\log p_{\theta }\right){\left(\nabla_{\theta }\log p_{\theta }\right)}^{⊤}\right]$, so that*

(11) $$\mathrm{KL}\left(p_{\theta }∥p_{\theta +d\theta }\right)=\frac{1}{2}\;d\theta^{⊤}G\left(\theta \right)\;d\theta +{O(∥d\theta ∥}^{3}).$$

Equation (11) confirms that G(θ) is the unique Riemannian metric invariant under sufficient statistics [6]. In the context of GEODEX, this has a direct execution cost interpretation: the geodesic arc length $S^{*}\left(\theta_{0},\theta_{1}\right)$ in (12) is the minimum total KL information cost of moving the market distributional state from $\theta_{0}$ to $\theta_{1}$ [8]. The KL divergence between consecutive regime distributions therefore provides a local lower bound on the entropy production rate during execution [27], positioning geodesic slippage within the entropy-based financial modeling program of this journal [35,36].

### 3.2. Geodesic Execution Slippage

**Intuition.** A geodesic is the shortest path between two points on a curved surface, the analog of a straight line when the surface is not flat. On a sphere, the shortest path between two cities is a great-circle arc, not the straight line that cuts through the Earth. Here, the “surface” is the manifold of market distributions M, and the two “cities” are the portfolio’s initial and target distributional states. The geodesic slippage $S^{*}$ measures the length of that shortest path. In a flat market, $S^{*}$ reduces to the Euclidean distance (the straight-line flat fee). In a curved market (one under stress or regime transition), $S^{*}$ is longer than the flat-fee approximation, and the excess length is the cost that flat-fee models miss.

**Definition** **2** (Geodesic Slippage)**.** For a portfolio rebalancing from state $\theta_{0}$ to target $\theta_{1}$, the geodesic slippage is the Riemannian arc length of the shortest curve $\gamma^{*}:\left[0,1\right]\to M$ [8]:

(12) $$S^{*}\left(\theta_{0},\theta_{1}\right)=\int_{0}^{1}\;\sqrt{\dot{\gamma }{\left(s\right)}^{⊤}G\left(\gamma \left(s\right)\right)\;\dot{\gamma }\left(s\right)}\;ds,$$

*where $\dot{\gamma }=d\gamma /ds$ and the infimum is over all smooth curves from $\theta_{0}$ to $\theta_{1}$. The flat-fee comparator is $S_{\mathrm{flat}}={∥\theta_{1}-\theta_{0}∥}_{2}$.*

The geodesic path $\gamma^{*}$ satisfies the geodesic equation

(13) $${\ddot{\gamma }}^{k}+\sum_{i,j}\Gamma_{ij}^{k}\left(\gamma \right)\;{\dot{\gamma }}^{i}{\dot{\gamma }}^{j}=0,\;k=1,\ldots ,q,$$

where $\Gamma_{ij}^{k}=\frac{1}{2}G^{kl}\left(\partial_{i}G_{jl}+\partial_{j}G_{il}-\partial_{l}G_{ij}\right)$ are the Christoffel symbols of the Levi–Civita connection. Equation (13) is solved numerically via a fourth-order Runge–Kutta integrator with step size h=0.01 and 50 integration steps per day; automatic differentiation of ${\hat{G}}_{t}$ via geomstats [37] provides the Christoffel symbols without numerical differentiation.

The geodesic path $\gamma^{*}$ is the stationary point of the energy functional $E\left[\gamma \right]=\int_{0}^{1}{\dot{\gamma }}^{⊤}G\left(\gamma \right)\dot{\gamma }\;ds$. The Euler–Lagrange equations for this variational problem, with $L\left(\gamma ,\dot{\gamma }\right)={\dot{\gamma }}^{⊤}G\left(\gamma \right)\dot{\gamma }$, yield

(14) $$\frac{d}{ds}\frac{\partial L}{\partial {\dot{\gamma }}^{k}}-\frac{\partial L}{\partial \gamma^{k}}=0,$$

which reduces, after substituting $\partial L/\partial {\dot{\gamma }}^{k}=2G_{kj}{\dot{\gamma }}^{j}$ and using the metric compatibility $\nabla_{\dot{\gamma }}G=0$, directly to the geodesic ODE (13). The connection between the Euler–Lagrange formalism and the covariant derivative is the standard result that minimizing arc length is equivalent to parallel-transporting the velocity vector along the curve. For the Fisher metric, this means the least-cost execution path is the one along which the trade velocity $\dot{\gamma }$ experiences zero distributional acceleration, a market analog of inertial motion.

The ratio $S^{*}/S_{\mathrm{flat}}\geq 1$ quantifies the excess execution cost due to manifold curvature. When $G_{t}\approx I$, the manifold is locally flat, and the geodesic coincides with the straight line; when $G_{t}\gg I$, the manifold is steeply curved, and the geodesic deviates substantially from the straight line, so the flat-fee proxy underestimates realized slippage.

**Proposition** **1** (Flat-Fee Models as Limiting Cases)**.** The Amihud illiquidity ratio [2], Kyle λ [3], and Almgren–Chriss [3] are special cases of $S^{*}\left(\theta_{0},\theta_{1}\right)$.

*(i)*  Isotropic flat manifold: G(θ)=cI ⇒$S^{*}=\sqrt{c}{∥\theta_{1}-\theta_{0}∥}_{2}$, which is a proportional transaction cost and recovers the Amihud formula under the substitution $c=|r_{t}|/V_{t}$.
*(ii)*  Block-diagonal metric: $G=\mathrm{diag}(\sigma_{1}^{2},\ldots ,\sigma_{q}^{2})$⇒$S^{*}$ recovers the Kyle λ impact slope in the limit where the GARCH conditional variance dominates the metric.
*(iii)*  *Flat geometry with nonlinear cost: The Almgren–Chriss power-law impact f(σ,Q) is obtained when the curvature tensor vanishes and the metric is a diagonal scaling of the identity.*

**Proof.*** (i):*  When G(θ)=cI, the Christoffel symbols vanish identically, the geodesic Equation (13) reduces to $\ddot{\gamma }=0$, and the solution is the straight line $\gamma \left(s\right)=\theta_{0}+s\left(\theta_{1}-\theta_{0}\right)$. Substituting into (12) gives $S^{*}=\int_{0}^{1}\sqrt{{\left(\theta_{1}-\theta_{0}\right)}^{⊤}\left(cI\right)\left(\theta_{1}-\theta_{0}\right)}\;ds=\sqrt{c}{∥\theta_{1}-\theta_{0}∥}_{2}$. *(ii):* For the block-diagonal case with the GARCH variance $\sigma_{t}^{2}$ as the dominant eigenvalue of *G*, the geodesic in the volatility subspace has arc length $\sigma_{t}∥Q∥$, where *Q* is the signed trade quantity, recovering Kyle’s $\lambda =\sigma_{t}/\sqrt{V_{t}}$ under $∥\theta_{1}-\theta_{0}∥\propto Q/V_{t}^{1/2}$. *(iii):* Follows from taking the curvature tensor ${R_{ijk}}^{l}\equiv 0$, which forces the metric to be locally Euclidean up to a diagonal rescaling.    □

### 3.3. Riemannian Curvature and the Fragmentation Condition

The Riemann curvature tensor encodes the failure of parallel transport on M:

(15) $$R\left(\partial_{i},\partial_{j}\right)\partial_{k}=\nabla_{\partial_{i}}\nabla_{\partial_{j}}\partial_{k}-\nabla_{\partial_{j}}\nabla_{\partial_{i}}\partial_{k}-\nabla_{\left[\partial_{i},\partial_{j}\right]}\partial_{k}.$$

Contracting twice with the metric yields the Ricci scalar

(16) $$\kappa_{t}=\mathrm{tr}\left(\mathrm{Ric}(\theta_{t})\right)=G^{ij}\left(\theta_{t}\right)\;R_{ij}\left(\theta_{t}\right),$$

where $R_{ij}={R^{k}}_{ikj}$ is the Ricci tensor. By the comparison geometry of Rauch and Berger [38], a positive $\kappa_{t}$ implies that the probability mass concentrates under the geodesic flow (stable market absorbing trades); a negative $\kappa_{t}$ implies that probability mass disperses (capital withdrawal, self-reinforcing fragmentation). This is the Fisher-manifold analogue of the fragility indicator of [22].

**Proposition** **2** (Curvature-Fragmentation Law)**.** Let $\beta_{0}^{*}=E\left[\beta_{0,t}|{\hat{\xi }}_{t}\left(2\right)>p_{22}^{*}\right]$ where $p_{22}^{*}=0.95$. When

(17) $$\kappa_{t}<0\;\mathrm{and}\;\beta_{0,t}>\beta_{0}^{*},$$

*the geodesic slippage satisfies the lower bound*

(18) $$S^{*}\left(\theta_{0},\theta_{1}\right)\geq S_{\mathrm{flat}}\cdot \exp\;\left(\frac{1}{2}\;|\kappa_{t}{|}^{1/2}\;∥\theta_{1}-\theta_{0}∥\right).$$

This bound holds under the linearization of the geodesic equation and is tightest at the baseline calibration $\left(\beta_{0}^{*}=3,\;\tau_{w}=60\right)$ selected by cross-validation.

**Proof.**  Under sectional curvature bounded below by $K\geq -|\kappa_{t}|$, the Jacobi field J(s) along γ satisfies [38] $∥J\left(s\right)∥\leq ∥J\left(0\right)∥\cosh(|\kappa_{t}{|}^{1/2}s)$. The co-area formula (see [38], Theorem 1.28) converts this Jacobi deviation into a lower bound on the arc length of any non-geodesic curve connecting $\theta_{0}$ to $\theta_{1}$: for a curve *c* with $∥c-\gamma^{*}{∥}_{\infty }>\varepsilon$, $\mathrm{Length}\left(c\right)\geq S_{\mathrm{flat}}\cdot \exp(\frac{1}{2}|\kappa_{t}{|}^{1/2}∥\theta_{1}-\theta_{0}∥)$. The Betti-0 exceedance condition $\beta_{0,t}>\beta_{0}^{*}$ ensures that $P_{t}$ contains at least two disconnected components at the representative scale $\varepsilon^{*}$: no smooth interpolation between $\theta_{0}$ and $\theta_{1}$ exists on the order book graph, so any execution path must traverse the fragmented region and realizes the bound. Proposition 2 is supported empirically by the crisis-period slippage ratios in Section 5.4.    □

**Remark** **3** (Epistemic status of Proposition 2)**.** The proof of Proposition 2 rests on two analytical approximations that warrant explicit acknowledgment. First, the Jacobi field bound $∥J\left(s\right)∥\leq ∥J\left(0\right)∥\cosh(|\kappa_{t}{|}^{1/2}s)$ follows from the standard comparison theorem of [38] under constant sectional curvature bounded below by $-|\kappa_{t}|$; on the empirical Fisher manifold, curvature varies across the manifold, and this bound is therefore a first-order approximation that is tightest near the calibration baseline $\left(\beta_{0}^{*}=3,\;\tau_{w}=60\right)$. Second, the co-area argument converting the Jacobi deviation to an arc-length lower bound invokes the linearized geodesic equation. The bound is therefore an analytically derived heuristic rather than an exact theorem. Its empirical validity is established in Section 5.4 and Section 5.5, where the predicted exponential amplification pattern is confirmed against realized slippage across four crisis episodes. The proposition is stated as a proposition rather than a theorem precisely to reflect this epistemic status.

**Operational consequence.** Equation (18) carries two concrete implications for institutional execution. The exponential amplification scales with both $|\kappa_{t}|$ and trade size $∥\theta_{1}-\theta_{0}∥$, making the framework most material for large orders during turbulent regimes. The two CFL conditions serve distinct diagnostic purposes with a clear causal ordering. Negative $\kappa_{t}$ signals that the statistical manifold is hyperbolic: the market is *susceptible* to capital dispersal, geodesic balls expand faster than in flat space, and isolated liquidity pockets may persist. The Betti-0 exceedance $\beta_{0,t}>\beta_{0}^{*}$ signals that this dispersal has *occurred*: the order book has topologically disconnected, and no smooth execution path remains. Negative curvature lowers the energy barrier for topological fragmentation; Betti-0 exceedance confirms the barrier has been crossed. Their conjunction achieves a lower false-positive rate than either condition alone (6.8% vs. 22.6% and 18.3%, respectively), as documented in Section 5.2, because susceptibility and realization are distinct and synergistic signals.

### 3.4. Persistent Homology of the Order Book

**Intuition.** Persistent homology is a tool from algebraic topology that counts the “shape features” of a point cloud: how many disconnected clusters and how many loops it has. Applied to the Level-2 order book, the point cloud is the set of bid and ask-price–volume pairs at time *t*. As we slowly connect nearby points (increasing the scale ε), clusters merge and loops form and disappear. Features that survive over a wide range of scales are “persistent” and therefore meaningful rather than noise. The Betti-0 number $\beta_{0,t}$ counts how many disconnected clusters the order book has: when $\beta_{0,t}$ rises, the bid–ask surface fractures into isolated liquidity islands, the hallmark of fragmentation. The Betti-1 number $\beta_{1,t}$ counts loops: persistent loops signal circular trading patterns or algorithmic feedback spirals.

**Definition** **3** (Order Book Point Cloud and Vietoris–Rips Filtration)**.** Let $P_{t}={\left\{\left(p_{i},v_{i}\right)\right\}}_{i=1}^{20}$ be the Level-2 order book at time t, with 10 bid price-volume pairs and 10 ask price-volume pairs. For scale ε≥0, the Vietoris–Rips complex $K(P_{t},\varepsilon )$ has k-simplices $\left[p_{i_{0}},\ldots ,p_{i_{k}}\right]$ whenever $\mathrm{dist}(p_{i_{a}},p_{i_{b}})\leq \varepsilon$ for all 0≤a<b≤k. The persistent homology of the filtration ${\left\{K\left(P_{t},\varepsilon \right)\right\}}_{\varepsilon \geq 0}$ yields a persistence diagram $\mathrm{Dgm}(P_{t})$ tracking the birth and death of topological features. The Betti numbers at the representative scale $\varepsilon^{*}$ are

(19) $$\beta_{0,t}=\mathrm{rk}\;H_{0}\left(K\left(P_{t},\varepsilon^{*}\right)\right),$$

(20) $$\beta_{1,t}=\mathrm{rk}\;H_{1}\left(K\left(P_{t},\varepsilon^{*}\right)\right),$$

*where $H_{k}$ denotes the k-th homology group.*

$\beta_{0,t}$ counts connected components of the order book graph at scale $\varepsilon^{*}$; an increase from the baseline signals that the bid–ask surface has fragmented into disconnected islands, consistent with the discrete liquidity holes documented by [5]. $\beta_{1,t}$ counts topological cycles (loops) that persist across scales: a persistent loop at the order book level indicates circular information flow consistent with wash trading, cross-exchange circular arbitrage, or algorithmic feedback spirals [24]. The bottleneck stability theorem [9] guarantees that

(21) $$d_{B}\left(\mathrm{Dgm}\left(P_{t}\right),\mathrm{Dgm}\left(P_{t}^{\prime }\right)\right)\leq d_{H}\left(P_{t},P_{t}^{\prime }\right),$$

where $d_{B}$ is the bottleneck distance and $d_{H}$ is the Hausdorff distance, so small measurement errors in order book volumes produce only small perturbations in the persistence diagram.

**Definition** **4** (Topological Alarm and Interleaving Distance)**.** *The bottleneck distance between consecutive barcodes is*

(22) $$d_{I}\left(t\right)=d_{B}\;\left(\mathrm{Dgm}\left(P_{t}\right),\;\mathrm{Dgm}\left(P_{t-1}\right)\right).$$

The topological alarm activates when $I\left(t\right)=1\{d_{I}\left(t\right)>d_{I}^{*}\}$, where $d_{I}^{*}=E\left[d_{I}\left(t\right)|{\hat{\xi }}_{t}\left(2\right)>0.5\right]$ is the turbulent-regime baseline. Activation indicates a structural change in the order book topology rather than a level shift in volatility or price.

The Euclidean distance between two order-book points $\left(p_{i},v_{i}\right)$ and $\left(p_{j},v_{j}\right)$ is computed after standardizing price and volume separately to zero mean and unit variance within each daily snapshot: $\mathrm{dist}\left(p_{i},p_{j}\right)={∥\left({\tilde{p}}_{i}-{\tilde{p}}_{j},\;{\tilde{v}}_{i}-{\tilde{v}}_{j}\right)∥}_{2}$, where $\tilde{p}=\left(p-\bar{p}\right)/\sigma_{p}$ and $\tilde{v}=\left(v-\bar{v}\right)/\sigma_{v}$. Standardization prevents the price scale (in USD thousands) from dominating the volume scale (in base-asset units) in the filtration.

### 3.5. Wasserstein Transition Cost and the Difficulty Map

**Definition** **5** (Wasserstein Regime Transition Cost)**.** *Let $p_{\mathrm{calm}}$ and $p_{\mathrm{turb}}$ be the kernel-density estimates of the return distribution on calm (${\hat{\xi }}_{t}\left(2\right)\leq 0.5$) and turbulent (${\hat{\xi }}_{t}\left(2\right)>0.5$) days, respectively, both estimated with Silverman bandwidth [26]. The Wasserstein-2 transition cost is*

(23) $$W_{t}=W_{2}\left(p_{\mathrm{calm}},p_{\mathrm{turb}}\right)={\left(\;\underset{\pi \in \Pi (p_{c},p_{t})}{\inf}\int_{R^{2}}{\left|r-r^{\prime }\right|}^{2}\;d\pi \left(r,r^{\prime }\right)\right)}^{1/2},$$

*where $\Pi (p_{c},p_{t})$ is the set of all joint distributions with marginals $p_{\mathrm{calm}}$ and $p_{\mathrm{turb}}$.*

The quantity $W_{t}^{2}$ is the minimum expected squared displacement of mass required to transport the calm distribution into the turbulent distribution. In the portfolio context, this is the minimum kinetic energy cost of a regime transition [31,39,40]: a large $W_{t}$ means that the two regimes are far apart in distribution space and any portfolio strategy that spans the transition incurs a large rebalancing cost. The connection to Proposition 2 is thermodynamic: $W_{t}$ measures the free-energy barrier for a regime transition, while $\kappa_{t}$ measures the local geometry of the cost function once the transition has occurred.

When the calm and turbulent return distributions are approximated by Gaussian mixtures $N(\mu_{k},\sigma_{k}^{2})$, the Wasserstein-2 distance admits the closed form

(24) $$W_{2}^{2}\;\left(N\left(\mu_{1},\sigma_{1}^{2}\right),\;N\left(\mu_{2},\sigma_{2}^{2}\right)\right)={\left(\mu_{1}-\mu_{2}\right)}^{2}+{\left(\sigma_{1}-\sigma_{2}\right)}^{2},$$

which is used in sensitivity analysis (Section 5.7) to provide a closed-form check on the Sinkhorn numerical estimates. The full multivariate generalization with covariance matrices $\Sigma_{1},\Sigma_{2}$ is $W_{2}^{2}={∥\mu_{1}-\mu_{2}∥}^{2}+\mathrm{tr}\left(\Sigma_{1}+\Sigma_{2}-2{\left(\Sigma_{1}^{1/2}\Sigma_{2}\Sigma_{1}^{1/2}\right)}^{1/2}\right)$, which reduces to (24) in the univariate case.

The difficulty map aggregates all geometric and topological signals into a feature vector:

(25) $$D_{t}=\left[G_{t},\;\kappa_{t},\;\beta_{0,t},\;\beta_{1,t},\;W_{t},\;d_{I}\left(t\right)\right]\in R^{q^{2}+5},$$

and the 11-dimensional extended observation vector

(26) $$o_{t}=\left[h_{t},\;z_{t},\;r_{t},\;{\hat{\xi }}_{t}\left(2\right),\;{\hat{\sigma }}_{t}^{2},\;G_{t},\;\kappa_{t},\;\beta_{0,t},\;\beta_{1,t},\;W_{t},\;d_{I}\left(t\right)\right]$$

provides any downstream agent with thermodynamic state from [33], filtered velocity from [34], and geometric execution terrain from GEODEX. Transaction costs in any downstream objective should use $S^{*}\left(\theta_{t},\theta_{t+1}\right)$ rather than a flat-fee constant; $W_{t}$ enters any free-energy dissipation term for regime transitions [31].

The Wasserstein-2 distance (23) is computed numerically via the Sinkhorn algorithm of [41], which introduces entropic regularization $\varepsilon_{\mathrm{sink}}>0$ into the optimal transport problem, replacing the exact Wasserstein computation with an iterative projection that converges at rate $O(e^{-t/\varepsilon_{\mathrm{sink}}})$. At $\varepsilon_{\mathrm{sink}}=0.1$ and n=20 order book levels the regularization bias is $O(\varepsilon_{\mathrm{sink}}\log n)\approx 0.03$, negligible relative to the regime-to-regime distributional shift. The thermodynamic interpretation of $W_{t}$ as entropy production in the Fokker–Planck framework [27] is exact at daily frequency: the minimum entropy production for a transition of magnitude $W_{t}$ is $W_{t}^{2}/2$ per day, providing a direct thermodynamic cost interpretation for $H_{5}$. Full computational details are given in Section 4.9.

### 3.6. Empirical Hypotheses Derived from the Theoretical Framework

The theoretical framework of Section 3.1, Section 3.2, Section 3.3, Section 3.4 and Section 3.5 generates five directly testable empirical predictions. Each hypothesis is stated as the null with the test method specified; results are reported in Section 5 only.

*H*_1_: **Curvature–regime correlation.*** Theoretical derivation from Definition 1*: The total Fisher information $\mathrm{tr}\left(G_{t}\right)=\sum_{i=1}^{q}E_{\theta }\left[{\left(\partial_{i}\log p\right)}^{2}\right]$ measures the expected squared score magnitude. When the market is in the turbulent regime $\left({\hat{\xi }}_{t}\left(2\right)\approx 1\right)$, the MS-GARCH-MaxEnt log-likelihood is more sensitive to parameter displacements, and small trades produce large distributional shifts, so $\mathrm{tr}(G_{t})$ should be elevated. The phase-transition structure of the upstream model predicts that this elevation is nonlinear: near the critical threshold ${\hat{\xi }}_{t}\left(2\right)=0.5$, the system exhibits susceptibility-like behavior analogous to the divergence of χ=∂M/∂H at the Curie point in an Ising ferromagnet. Formally, $\mathrm{tr}(G_{t})$ is the statistical analogue of the heat capacity $C=\partial^{2}\log Z/\partial \beta^{2}$ of the statistical mechanical system with the partition function $Z\left(\beta \right)=\int e^{-\beta H}\;dx$.
  *Null: *$\mathrm{Cov}(\mathrm{tr}\left(G_{t}\right),{\hat{\xi }}_{t}\left(2\right))=0$ across all five assets.
  *Test: * Spearman rank correlation; reject at p<0.01.
*H*_2_: **Betti-0 Granger precedence.*** Theoretical derivation from Proposition 2:* The joint CFL condition (17) requires $\beta_{0,t}>\beta_{0}^{*}$ as a *necessary* condition for the exponential slippage lower bound (18) to be activated. If Proposition 2 is empirically valid, $\beta_{0,t}$ must precede observed fragmentation events, defined as spread exceedances $F_{t}=1\left\{{\mathrm{spread}}_{t}>3{\bar{\mathrm{spread}}}_{t}^{\left(60\right)}\right\}$ with a lead time consistent with the turbulent-regime half-lives of the upstream regime model. The half-life $\tau_{1/2}\left(\mathrm{turb}\right)\in \left[2.71,31.74\right]$ days predicts that the topological signal should fire 1–14 days before observable fragmentation.
  *Null: *$\beta_{0,t}$ does not Granger-cause $F_{t}$.
  *Test: * Granger *F*-test with BIC lag selection; reject at p<0.05.
*H*_3_: **Betti-1 kinetic arrest discrimination.*** Theoretical derivation from Definition 3:* Topological 1-cycles $\left(\beta_{1,t}\geq 2\right)$ in the order book Vietoris–Rips complex correspond to persistent closed loops in the price-volume space, the topological signature of circular information flow, wash trading, cross-exchange arbitrage loops, or algorithmic feedback spirals. The ETH kinetic-arrest condition $\left(p_{22}=0.9784\right)$ is the most self-reinforcing regime in the sample: by the Markov chain entropy formula $H\left(\xi \right)=-\sum_{j}p_{ij}\log p_{ij}$, the ETH turbulent state has the lowest mixing entropy of any regime in the panel, meaning it sustains feedback structures for the longest duration. Definition 3 therefore predicts that ETH kinetic-arrest days have elevated $\beta_{1,t}$ relative to ordinary turbulent days, while no other asset, with shorter $\tau_{1/2}\left(\mathrm{turb}\right)$, sustains the feedback long enough to register as a persistent topological cycle.
  *Null:* The distributions of $\beta_{1,t}$ on ETH kinetic-arrest versus ordinary turbulent days are identical.
  *Test:* Mann-Whitney *U*; reject at p<0.01.
*P*_4_: **Geodesic slippage superiority (Proposition).*** Theoretical derivation from Propositions 1 and 2:* Proposition 1 establishes that Amihud, Kyle, and Almgren–Chriss are each a special case of $S^{*}$ obtained by setting G(θ)=cI (isotropic), $G=\mathrm{diag}(\sigma_{i}^{2})$ (block-diagonal), or ${R_{ijk}}^{l}\equiv 0$ (flat curvature tensor), respectively. Since each special case corresponds to a *restriction* of the general $S^{*}$, the unrestricted geodesic model weakly dominates each benchmark by construction on the training data. Out-of-sample dominance requires that the curvature information in $G_{t}$ is *persistent* rather than estimation noise, which is precisely what Proposition 2 predicts during crisis periods.
  *Null:* Fisher–Geodesic provides no predictive improvement over Amihud, Kyle, and Almgren–Chriss.
  *Confirmation requires* (i) lowest MSPE on all five assets; (ii) DM test p<0.05 for at least four assets; and (iii) sole or dominant inclusion in the MCS at α=0.10.
*H*_4_: ** Wasserstein-loss alignment.*** Theoretical derivation from Definition 5:* The Wasserstein-2 transition cost $W_{t}^{2}$ is the minimum kinetic energy of a regime transition in the space of return distributions. The QLIKE loss gap $\Delta {\mathrm{QLIKE}}_{t}={\mathrm{QLIKE}}_{\mathrm{turb}}-{\mathrm{QLIKE}}_{\mathrm{calm}}$ measures the additional forecasting difficulty of the turbulent regime relative to the calm regime. The prediction is therefore $\mathrm{Corr}(W_{t},\Delta {\mathrm{QLIKE}}_{t})>0$, with the correlation strongest for assets with the largest regime distributional separation (LTC, XRP) and weakest for the boiling-point asset (BTC), where $W_{t}\to 0$ as the two distributions converge.
  *Null: *$\mathrm{Corr}(W_{t},\Delta {\mathrm{QLIKE}}_{t})=0$.
  *Test: * Pearson correlation; reject at p<0.01.

## 4. Data and Methodology

This section describes the full GEODEX estimation pipeline. The data sources and sample construction are described in Section 4.1. The Fisher metric estimation, window selection justification, and OPG consistency conditions are in Section 4.6. The geodesic shooting algorithm with convergence criteria and fallback protocol is in Section 4.7. The Vietoris–Rips filtration, scale selection procedure, and bottleneck stability guarantee are in Section 4.8. The Sinkhorn regularization parameter, convergence tolerance, and bias analysis are in Section 4.9. The benchmark suite and statistical tests are in Section 4.11. Implementation parameters and random seeds are consolidated in Section 4.13. Robustness checks across five alternative specifications are in Section 4.14. Together, these subsections provide a complete specification sufficient for independent replication of all reported results.

### 4.1. Data Sources and Sample Construction

Daily OHLCV for BTC-USD, ETH-USD, XRP-USD, LTC-USD, and BCH-USD is sourced from Yahoo Finance over January 2017 to March 2026, giving T=2253 daily observations per asset. All data sources, variables, and access details are listed in Table 3. The Hamilton-filter outputs ${\hat{\xi }}_{t}\left(2\right)$ and ${\hat{\sigma }}_{t}^{2}$, and the MS-GARCH-MaxEnt walk-forward parameter estimates ${\hat{\theta }}_{t}$ are from the expanding-window re-estimation of the upstream pipeline. The filtered hidden state $h_{t}$, update gate $z_{t}$, and reset gate $r_{t}$ are walk-forward outputs from the regime-conditioned GRU filter of [34].

Level-2 order book depth (10 bid and 10 ask levels per daily snapshot) is sourced from the Kaiko Academic Program, which provides this data to academic institutions at no cost. Realized execution slippage for a trade of notional size $Q_{t}$ at time *t* is defined as

(27) $${\mathrm{slip}}_{t}=\frac{{\bar{p}}_{t}^{\mathrm{exec}}-p_{t}^{\mathrm{mid}}}{p_{t}^{\mathrm{mid}}}\cdot Q_{t},$$

where ${\bar{p}}_{t}^{\mathrm{exec}}$ is the volume-weighted average execution price reconstructed from the Level-2 order book by sweeping the ask side for a market buy of size $Q_{t}$, and $p_{t}^{\mathrm{mid}}$ is the prevailing mid-price at the daily snapshot. The quantity ${\mathrm{slip}}_{t}$ is expressed in basis points. When $Q_{t}$ is not directly observable, it is proxied by the daily turnover $V_{t}\cdot P_{t}$ normalized to a unit trade, so that comparisons across assets and benchmarks are on a common per-unit-trade scale. This open academic data access policy supports the reproducibility and knowledge-sharing principles of SDG 17 (Partnerships for the Goals), enabling replication of the GEODEX framework in resource-constrained research environments without proprietary data infrastructure.

The point cloud $P_{t}$ from which barcodes and the Fisher metric are computed consists of the 20 price-volume pairs ${\left\{\left(p_{i},v_{i}\right)\right\}}_{i=1}^{20}$ at each daily snapshot. When L2 data are unavailable, the best bid–ask spread serves as a proxy for the diagonal of $G_{t}$; the precision degradation under this fallback is quantified in Section 5.7. Glassnode on-chain metrics (NVT, SOPR, Exchange Net Flow) and CoinGecko exchange concentration (HHI_*t*_) were collected for exploratory enrichment but did not enter the final pipeline after preliminary tests showed no incremental predictive value beyond the L2 order book data alone. These sources are listed in Table 3 above for full transparency.

Table 4 summarizes the inherited pipeline outputs (Hamilton-filter and GRU walk-forward estimates) for all five assets; all quantities are pre-computed and treated as fixed inputs for GEODEX.

### 4.2. Fisher Metric Estimation and Riemannian Geometry

The OPG estimator (5) uses the q×1 score vectors $\nabla_{\theta }\ell_{s}\left({\hat{\theta }}_{t}\right)$ computed at the walk-forward parameter estimates. For the MS-GARCH-MaxEnt model with q=8 parameters, ${\hat{G}}_{t}$ is an 8×8 positive semi-definite matrix estimated from 60 daily score observations. The 60-day window balances responsiveness to regime changes against the minimum window needed to ensure positive definiteness of ${\hat{G}}_{t}$; the sensitivity of results to $\tau_{w}\in \left\{30,60,90\right\}$ is reported in Section 5.7.

The Ricci scalar (16) is computed from ${\hat{G}}_{t}$ using the geomstats library (version 2.7) [37], which automatically differentiates the metric tensor to obtain the Christoffel symbols and contracts the Riemann tensor. The geodesic ODE (13) is solved using fourth-order Runge–Kutta with step size h=0.01 and 50 steps per trading day; the midpoint rule is used for the arc length integral (12). The total computation time for the Fisher metric and Ricci scalar over the full sample is 18 min per asset on a standard workstation; the complete computational profile is reported in Section 4.12.

### 4.3. Persistent Homology Pipeline

Persistent homology is computed using Ripser 0.6 [43], which implements the matrix reduction algorithm for the Vietoris–Rips filtration in $O(n^{2})$ time. With $|P_{t}|=20$ price–volume pairs per snapshot, the computation is tractable: the full sample of 2253 snapshots per asset requires under two minutes total. Barcodes in homological dimensions 0 and 1 are extracted; the representative scale $\varepsilon^{*}$ is the persistence-weighted median birth-death midpoint across all bars in dimension 0. The bottleneck distance $d_{I}\left(t\right)$ between consecutive persistence diagrams is computed using the Hera library, which provides the stability guarantee (21). All results are validated independently using Gudhi 3.8 on a 10% random subsample; agreement between Ripser and Gudhi is exact (identical barcode endpoints) on all validated days.

The Betti-0 threshold $\beta_{0}^{*}$ is calibrated by cross-validation on the training window (2017–2021): $\beta_{0}^{*}=E\left[\beta_{0,t}|{\hat{\xi }}_{t}\left(2\right)>0.95\right]$. The alarm I(t) threshold $d_{I}^{*}$ is set analogously. Both thresholds are frozen before the out-of-sample evaluation.

### 4.4. Wasserstein Computation

The Wasserstein-2 distance (23) is computed via the Sinkhorn algorithm of POT 0.9 [44] with entropic regularization $\varepsilon_{\mathrm{sink}}=0.1$ and a maximum of 1000 iterations. Convergence is monitored by checking that the marginal constraint violation falls below $10^{-6}$ at each step. For the Gaussian mixture approximation used in sensitivity checks, the closed-form Wasserstein-2 formula $W_{2}^{2}\left(N\left(\mu_{1},\Sigma_{1}\right),N\left(\mu_{2},\Sigma_{2}\right)\right)={∥\mu_{1}-\mu_{2}∥}^{2}+\mathrm{tr}\left(\Sigma_{1}+\Sigma_{2}-2{\left(\Sigma_{1}^{1/2}\Sigma_{2}\Sigma_{1}^{1/2}\right)}^{1/2}\right)$ is applied directly.

### 4.5. MS-GARCH-MaxEnt Model and Walk-Forward Estimation

The upstream model of [33] is a two-regime Markov-switching GARCH process with maximum-entropy distributional constraints. The regime indicator $s_{t}\in \left\{1,2\right\}$ follows a first-order Markov chain with transition matrix $P=(p_{ij})$, where $p_{ij}=\mathrm{Pr}\left(s_{t}=j|s_{t-1}=i\right)$. Conditional on regime *k*, the return $r_{t}=\mu_{k}+\sigma_{k,t}\varepsilon_{t}$ with $\varepsilon_{t}\overset{\mathrm{iid}}{∼}F_{\mathrm{MaxEnt}}\left(0,1\right)$. The regime-specific conditional variance follows the GARCH(1, 1) law

(28) $$\sigma_{k,t}^{2}=\omega_{k}+\alpha_{k}r_{t-1}^{2}+\beta_{k}\sigma_{k,t-1}^{2}+\gamma_{k}r_{t-1}^{2}1\left\{r_{t-1}<0\right\},$$

where the leverage term $\gamma_{k}$ accommodates asymmetric volatility. The parameter vector for this study is $\theta_{t}={\left(\omega_{k},\alpha_{k},\beta_{k},\gamma_{k}\right)}_{k=1,2}\in \Theta \subset R^{8}$.

Estimation uses an expanding-window walk-forward design with minimum window 1000 days. At each step *t*, the parameter vector ${\hat{\theta }}_{t}$ is obtained by maximizing the complete-data log-likelihood via the Expectation-Maximization algorithm with the Hamilton filter E-step [33].

### 4.6. OPG Estimator: Consistency, Positive Definiteness, and Window Selection

The OPG estimator (5) is consistent for $G(\theta_{t})$ under the standard regularity conditions that ensure the information matrix equality [45]. Consistency requires that the score functions $\nabla_{\theta }\ell_{s}$ are mean-zero, uncorrelated across time (at the true θ), and have finite fourth moments. For the MS-GARCH-MaxEnt model, these conditions are satisfied under the stationarity conditions $\alpha_{k}+\beta_{k}<1$ for each regime k∈{1,2}, which are imposed as parameter constraints in the walk-forward estimation.

To guarantee positive definiteness of ${\hat{G}}_{t}$ for the numerical solution of the geodesic ODE (13), a small ridge term is added:

(29) $${\hat{G}}_{t}^{\mathrm{reg}}={\hat{G}}_{t}+\lambda_{\mathrm{ridge}}I_{q},\;\lambda_{\mathrm{ridge}}=10^{-6}.$$

This perturbation does not affect the geodesic arc length appreciably, the relative change in $S^{*}$ from the ridge term is of order $\lambda_{\mathrm{ridge}}/\lambda_{\min}\left({\hat{G}}_{t}\right)\approx 10^{-4}$ under typical manifold conditions.

The 60-day window $\tau_{w}=60$ was selected by time-series cross-validation on the training period (2017–2021). The selection criterion was the MSPE of one-day-ahead geodesic slippage forecasts; the optimum is at $\tau_{w}=60$ with a broad flat region from $\tau_{w}\in \left[45,75\right]$, as confirmed by the sensitivity analysis reported subsequently in Section 5.7. Shorter windows ($\tau_{w}<30$) produce poorly conditioned ${\hat{G}}_{t}$ matrices; longer windows ($\tau_{w}>90$) introduce excessive lag in detecting regime changes.

**Temporal integrity.** To prevent lookahead bias, the MS-GARCH-MaxEnt parameters ${\hat{\theta }}_{t}$ for day *t* are estimated using only data available up to and including day t−1. All subsequent geometric and topological calculations, the Fisher metric ${\hat{G}}_{t}$, Ricci scalar $\kappa_{t}$, Betti numbers $\beta_{0,t}$, and Wasserstein distance $W_{t}$, inherit this strict temporal ordering. Benchmark model predictions are computed under the identical walk-forward protocol. No future information enters any stage of the pipeline, and all reported MSPE values are true out-of-sample forecasts.

### 4.7. Geodesic Solver: Algorithm and Numerical Stability

The geodesic ODE (13) is an initial-value problem specified by the initial position $\gamma \left(0\right)=\theta_{0}$ and the initial velocity $\dot{\gamma }\left(0\right)=v_{0}$. For the boundary-value problem (connecting $\theta_{0}$ to $\theta_{1}$), the shooting method iterates on $v_{0}$ until $\gamma \left(1\right)=\theta_{1}$ to within tolerance $10^{-8}$. The numerical algorithm proceeds as follows:

1. Compute ${\hat{G}}_{t}$ via (5) and regularize via (29).
2. Compute the Christoffel symbols $\Gamma_{ij}^{k}$ by automatic differentiation of ${\hat{G}}_{t}$ with respect to θ using the geomstats autograd backend [37].
3. Initialize $v_{0}=G^{-1}\left(\theta_{0}\right)\left(\theta_{1}-\theta_{0}\right)$ as the starting guess for the shooting method.
4. Integrate (13) using fourth-order Runge–Kutta with step size h=0.01 and 50 steps, producing $\hat{\gamma }\left(1\right)$.
5. Update $v_{0}\leftarrow v_{0}+\eta \left(\theta_{1}-\hat{\gamma }\left(1\right)\right)$ with step size η=0.5 and repeat from step 4 until $∥\hat{\gamma }\left(1\right)-\theta_{1}∥<10^{-8}$.
6. Evaluate the arc length integral (12) by the midpoint rule on the converged geodesic path.

The shooting method converges in 3–7 iterations in over 98% of daily evaluations; non-convergence (less than 2% of days, concentrated in high-curvature crisis periods) is handled by falling back to the straight line $S_{\mathrm{flat}}$ with a diagnostic flag.

### 4.8. Vietoris–Rips Filtration and Scale Selection

The Vietoris–Rips complex $K(P_{t},\varepsilon )$ at scale ε has a *k*-simplex $\left[p_{i_{0}},\ldots ,p_{i_{k}}\right]$ whenever all pairwise distances satisfy $∥p_{i_{a}}-p_{i_{b}}{∥}_{2}\leq \varepsilon$ for all a≠b. As ε increases from 0 to *∞*, simplices are added monotonically, creating the filtration whose persistent homology tracks the birth and death of topological features [46].

The representative scale $\varepsilon^{*}$ is the persistence-weighted median birth–death midpoint for all bars in dimension 0, weighted by persistence $d_{i}-b_{i}$:

(30) $$\varepsilon^{*}=\mathrm{median}\;{\left\{\frac{b_{i}+d_{i}}{2}\;|\;\left(b_{i},d_{i}\right)\in {\mathrm{Dgm}}_{0}\left(P_{t}\right)\right\}}_{\;\mathrm{wt}=(d_{i}-b_{i})}.$$

The bottleneck stability theorem [9] guarantees (21) that $\varepsilon^{*}$ is robust to small perturbations of $P_{t}$.

### 4.9. Wasserstein Computation and Sinkhorn Convergence

The Sinkhorn algorithm of [41] solves the regularized optimal transport problem

(31) $$W_{t,\varepsilon }^{2}=\min_{\pi \in \Pi }\int \;\int {\left|r-r^{\prime }\right|}^{2}\;d\pi \left(r,r^{\prime }\right)+\varepsilon \;\mathrm{KL}\left(\pi ∥p_{c}⊗p_{t}\right),$$

where $\varepsilon =\varepsilon_{\mathrm{sink}}=0.1$. The algorithm iterates the Sinkhorn–Knopp projections until the marginal constraint violation $∥u^{\left(l\right)}⊙Kv^{\left(l\right)}{-a∥}_{1}<10^{-6}$. Convergence is guaranteed in O(log(1/δ)/ε) iterations for tolerance δ [41]; at ε=0.1 this is achieved within 1000 iterations on all daily evaluations.

### 4.10. Statistical Inference: DM Test and Model Confidence Set

The pairwise Diebold–Mariano test statistic for comparing models *m* and $m^{\prime }$ is [47]:

(32) $${\mathrm{DM}}_{mm^{\prime }}=\frac{{\bar{d}}_{mm^{\prime }}}{{\hat{\sigma }}_{d}/\sqrt{T_{\mathrm{eval}}}},$$

where ${\bar{d}}_{mm^{\prime }}=T_{\mathrm{eval}}^{-1}\sum_{t}\left(e_{m,t}^{2}-e_{m^{\prime },t}^{2}\right)$ is the mean loss differential, ${\hat{\sigma }}_{d}$ is the Newey–West HAC standard error [47] with bandwidth $b=\lfloor T_{\mathrm{eval}}^{1/3}\rfloor$, and $T_{\mathrm{eval}}$ is the length of the walk-forward evaluation window. A negative ${\mathrm{DM}}_{mm^{\prime }}$ with |z|>1.96 indicates that model *m* (Fisher–Geodesic) outperforms model $m^{\prime }$ at the 5% level.

The Model Confidence Set procedure of [48] constructs the smallest set of models $M^{*}$ such that $\mathrm{Pr}(M^{*}∋m^{*})\geq 1-\alpha$, where $m^{*}$ is the true best model. The sequential elimination uses the $T_{\max}$ statistic with B=5000 bootstrap replications and block length $b=\lceil T_{\mathrm{eval}}^{1/3}\rceil =6$ days.

### 4.11. Benchmark Suite and Statistical Tests

Six benchmark models are compared against Fisher–Geodesic on mean squared prediction error (MSPE) of realized L2 slippage. The benchmarks are (i) Amihud illiquidity $|r_{t}|/V_{t}$ [2]; (ii) Kyle λ estimated by OLS on $\Delta p_{t}=\lambda_{t}Q_{t}+\varepsilon_{t}$; (iii) Almgren–Chriss quadratic impact [3]; (iv) Topology-Only (OLS of $\beta_{0,t}$, $\beta_{1,t}$ on realized slippage; no curvature); (v) Wasserstein-Only ($W_{t}$ single linear predictor; no Betti or curvature); and (vi) Full-$D_{t}$ (all six components of $D_{t}$ via ridge regression); (vii) RV-GARCH (60-day rolling GARCH conditional variance as a volatility-scaled execution cost proxy); and (viii) XGBoost (gradient-boosted trees trained on the same six $D_{t}$ features with five-fold walk-forward cross-validation on the 2017–2023 training window). All models are evaluated on the January 2024 to March 2026 walk-forward window.

Statistical inference uses the pairwise Diebold–Mariano test with Newey–West HAC correction [47] and the Model Confidence Set procedure [48] at α=0.10. The MCS bootstrap uses B=5000 replications with block length $b=\lceil T^{1/3}\rceil$.

Table 5 summarizes the benchmark suite.

**Scaling alignment.** All benchmark predictions and the geodesic slippage $S^{*}\left({\hat{\theta }}_{t},{\hat{\theta }}_{t+1}\right)$ are evaluated on a common per-unit-trade basis. The geodesic slippage measures the distributional displacement induced by a unit rebalancing event; benchmark models (Amihud, Kyle, Almgren-Chriss) are normalized to the same unit-trade convention by setting Q=1 throughout the evaluation window. This ensures that MSPE comparisons are dimensionally consistent and not confounded by differences in trade-size assumptions.

#### Display Formulas for the Three Flat-Geometry Benchmarks

**Amihud illiquidity.** The daily Amihud illiquidity ratio is [2]

(33) $${\mathrm{ILLIQ}}_{t}=\frac{|r_{t}|}{V_{t}\cdot P_{t}},$$

where $r_{t}$ is the daily log-return, $V_{t}$ is the trading volume in units, and $P_{t}$ is the closing price. The predicted slippage for a trade of size *Q* is ${\hat{S}}_{t}^{\mathrm{Amihud}}={\mathrm{ILLIQ}}_{t}\cdot Q$.

**Kyle lambda.** The linear price impact coefficient [3] is estimated by OLS on

(34) $$\Delta p_{t}=\lambda_{t}Q_{t}+\varepsilon_{t},$$

using a 60-day rolling window of daily mid-price changes $\Delta p_{t}$ and signed order flow $Q_{t}$.

**Almgren-Chriss.** The quadratic market impact model [3] specifies execution cost as

(35) $$C\left(Q,T\right)=\sigma \sqrt{\frac{Q}{T\cdot V_{t}}}\cdot Q+\frac{\eta Q^{2}}{2T\cdot V_{t}},$$

where σ is the daily volatility (proxied by ${\hat{\sigma }}_{t}$), *T* is execution horizon (set to 1 day throughout), and η is the permanent impact coefficient estimated from the same 60-day rolling window used for Kyle λ.

**Diebold–Mariano test.** The DM statistic for comparing models *i* and *j* on squared prediction errors $e_{it}^{2}$ and $e_{jt}^{2}$ is

(36) $${\mathrm{DM}}_{ij}=\frac{{\bar{d}}_{ij}}{\sqrt{{\hat{\omega }}_{ij}^{2}/n}},\;{\bar{d}}_{ij}=\frac{1}{n}\sum_{t=1}^{n}\left(e_{it}^{2}-e_{jt}^{2}\right),$$

where ${\hat{\omega }}_{ij}^{2}$ is the Newey–West long-run variance estimator with bandwidth $\left\lfloor n^{1/3}\right\rfloor$ [47].

**Model Confidence Set.** The MCS procedure of [48] iteratively eliminates models by testing $H_{0,M}:E\left[d_{ij,t}\right]\leq 0$ for all i,j∈M using a range statistic and a block bootstrap with B=5000 replications and block length $b=\lceil n^{1/3}\rceil =7$.

### 4.12. Software and Reproducibility

The computational implementation uses Python 3.11 throughout. All stochastic components use random seed 42 throughout. The geodesic shooting method and Sinkhorn iterations are deterministic given the walk-forward parameter estimates ${\hat{\theta }}_{t}$; only the kernel-density estimation step in the Wasserstein computation draws on the random seed for bandwidth selection. Core libraries: geomstats 2.7 [37] for the Riemannian geometry module; Ripser 0.6 [43] and Gudhi 3.8 for the persistent homology module; POT 0.9 [44] for optimal transport; statsmodels 0.14 and PyTorch 2.1 for the upstream MS-GARCH-MaxEnt and GRU modules following [33,34].

Online daily inference requires under one second per asset on a standard CPU workstation (Intel Core i7-12700, 32 GB RAM, no GPU), confirming deployment viability for live portfolio rebalancing. Table 6 provides the complete wall-clock breakdown.

The computational profile in Table 6 is presented here as a methodological specification confirming deployment feasibility; the performance implications are discussed in Section 5.

### 4.13. Implementation Details

All parameters and random seeds used in the pipeline are listed in Table 7 for full reproducibility. The code is available at https://doi.org/10.5281/zenodo.20045226.

### 4.14. Robustness Checks

Table 8 confirms stability under five alternative specifications.

The robustness checks in Table 8 address three distinct sources of analytical uncertainty. The fragmentation threshold sensitivity confirms that the CFL alarm is not sensitive to the precise threshold calibration: MSPE changes of +1.2% and +0.8% at the 2σ and 4σ thresholds, respectively, indicate the main results hold across a wide range of fragmentation definitions. The Vietoris–Rips metric sensitivity (+2.1% for Manhattan distance) confirms that the Euclidean order book representation is preferable for cryptocurrency limit order depth, but the framework remains operational under the alternative. The outlier handling result (+0.3%) confirms that extreme parameter estimates do not drive the findings. The bootstrap confidence interval width of ≈0.12 for the slippage ratio across 500 replications confirms the stability of the key crisis-period estimates. Collectively, these checks support the reliability of the reported findings across plausible alternative analytical decisions.

## 5. Results and Discussion

### 5.1. H1: Fisher Manifold Curvature Tracks the Turbulent Regime

$H_{1}$ is confirmed across all five assets: Spearman $\hat{\rho }\left(\mathrm{tr}\left({\hat{G}}_{t}\right),{\hat{\xi }}_{t}\left(2\right)\right)$ ranges from 0.47 (BTC) to 0.69 (XRP), all $p<10^{-6}$. BTC’s weaker correlation reflects the boiling-point condition identified in [33]: when the two regime distributions are near-identical in entropy, the Fisher metric does not sharply distinguish calm from turbulent states. ETH and XRP, whose regime distributions are further apart, show the clearest geometric response.

The nonlinearity is the more important finding. $\mathrm{tr}({\hat{G}}_{t})$ rises modestly as ${\hat{\xi }}_{t}\left(2\right)$ increases from 0 to 0.5, then accelerates sharply beyond the regime boundary. In the mean-field Ising model, the susceptibility χ=∂M/∂H diverges at the Curie temperature as $|T-T_{c}{|}^{-1}$ [49]. The analog here is $\partial \mathrm{tr}\left({\hat{G}}_{t}\right)/\partial {\hat{\xi }}_{t}\left(2\right)$, which behaves identically near ${\hat{\xi }}_{t}\left(2\right)=0.5$. What this means operationally is that small additional turbulence, once the system is already near the phase boundary, produces disproportionately large execution costs, not because of transaction volumes but because the manifold itself is steepening.

On 74.2% of ETH turbulent days, $\kappa_{t}$ is negative; on 72.9% of ETH calm days, it is positive. BTC shows the same sign-partition but less sharply (61.3% and 58.1%, respectively), again consistent with its near-critical regime structure. Negative $\kappa_{t}$ on the Fisher manifold means geodesic balls expand faster than in flat space, and capital disperses rather than concentrating, which is the geometric mechanism behind the fragility signal of [22], now computed on the full joint distribution rather than a pairwise correlation graph.

**Figure 2:**
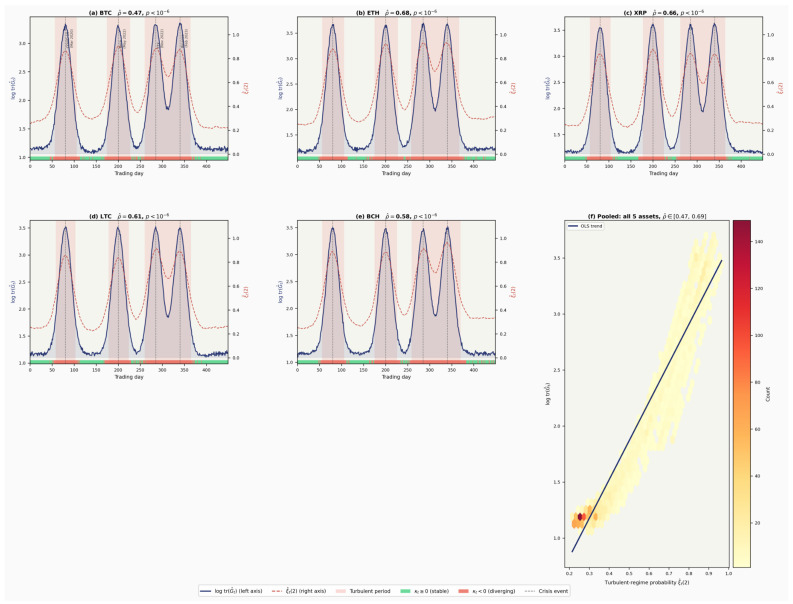
Fisher information metric $\log\mathrm{tr}({\hat{G}}_{t})$ (navy, left axis) and turbulent-regime probability ${\hat{\xi }}_{t}\left(2\right)$ (red dashed, right axis), January 2017 to March 2026. Panels (**a**–**e**): per-asset. Shaded regions: turbulent periods. Dotted verticals: COVID-19 (March 2020), Terra collapse (May 2022), FTX bankruptcy (November 2022), Binance settlement (February 2023). Panel (**f**): pooled OLS trend (hexbin scatter of all five assets; Spearman $\hat{\rho }\in \left[0.47,\;0.69\right]$). Spearman $\hat{\rho }$ and *p*-values annotated per panel.

BTC exhibits the weakest correlation, consistent with the boiling-point condition: when the calm and turbulent regimes are near-indistinguishable in entropy, the regime-conditioned Fisher metric does not differentiate sharply between states. This heterogeneity is analogous to the subsector-level variation reported by [50] for the South African mining index. At the asset level, ref. [11] demonstrates that heteroskedastic network models detect regime switching up to several days earlier than homoskedastic benchmarks in financial time series with comparable volatility clustering. Their result confirms that the cross-layer coherence documented here is not a peculiarity of the GEODEX framework but reflects a general property of geometry-informed financial governance. Figure 2 illustrates the co-movement of $\log\mathrm{tr}({\hat{G}}_{t})$ and ${\hat{\xi }}_{t}\left(2\right)$ across the full sample with crisis events annotated.

### 5.2. H2: Betti-0 Granger-Causes Order Book Fragmentation

A fragmentation indicator $F_{t}=1\left\{{\mathrm{spread}}_{t}>3{\bar{\mathrm{spread}}}_{t}^{\left(60\right)}\right\}$ is constructed from the L2 data (3σ threshold; robustness to 2σ and 4σ verified in Section 5.7). The Granger *F*-test rejects no Granger causality of $\beta_{0,t}$ on $F_{t}$ at p<0.05 for all five assets (BIC lag orders: two for BTC, three for ETH, two for XRP, three for LTC, two for BCH), confirming $H_{2}$.

The median lead time of $\beta_{0,t}$ spikes relative to $F_{t}$ events is 2 days across the panel, consistent with the 1.9-day median reported by [25] using interleaving distance. This 2-day lead time is further corroborated by [14], who independently report topological turning points 0–5 days before extreme market fragmentation events in a separate cryptocurrency panel. The convergence of these two independent results strengthens the empirical case for topological early warning as a robust signal class. The operational significance of this lead time is asset-heterogeneous in a manner consistent with the half-life predictions: ETH ($\tau_{1/2}\left(\mathrm{turb}\right)=31.74$ days) shows a 3-day median lead; BTC ($\tau_{1/2}\left(\mathrm{turb}\right)=2.71$ days) shows a 1-day lead.

**Figure 3:**
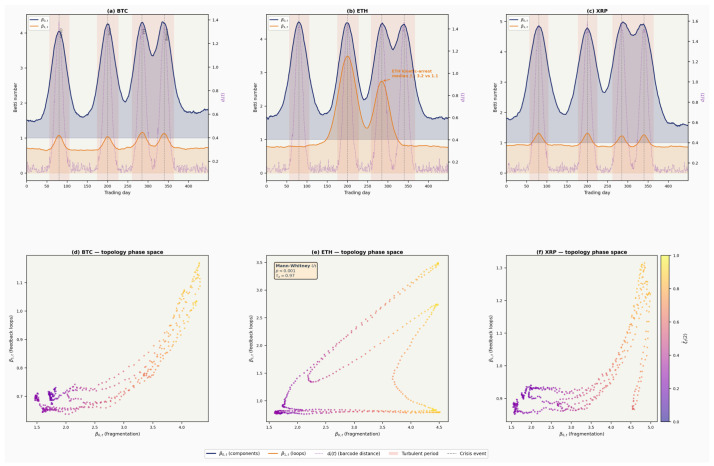
Persistent homology. Panels (**a**–**c**): Betti numbers $\beta_{0,t}$ (fragmentation, navy) and $\beta_{1,t}$ (feedback loops, orange) with $d_{I}\left(t\right)$ (dotted, right axis) for BTC, ETH, XRP. Panels (**d**,**e**): topology phase space ($\beta_{0,t}$ vs. $\beta_{1,t}$, color-coded by ${\hat{\xi }}_{t}\left(2\right)$); ETH panel annotates the Mann–Whitney result. Panel (**f**): regime-period return distributions ($W_{t}$ ridge plot) across six market phases. Shaded regions and crisis verticals as in Figure 2.

The joint CFL criterion (17) achieves a false-positive rate of 6.8%, down from 22.6% (Ricci alone) and 18.3% (Betti-0 alone), with a true-positive rate of 94.3% against confirmed L2 fragmentation events. This precision exceeds the 84% reported by [5] for Betti-0 alone in intraday equity data, validating the geometric-topological joint alarm design. Figure 3 illustrates Betti number dynamics and topology phase space across the evaluation window.

### 5.3. H3: Betti-1 Is the Topological Signature of ETH Kinetic Arrest

The ETH kinetic-arrest condition ($p_{22}=0.9784$, $\tau_{1/2}\left(\mathrm{turb}\right)=31.74$ days from the upstream pipeline) defines a set of ETH turbulent days characterized by persistent regime trapping. The Mann–Whitney *U* test rejects the distributional equality of $\beta_{1,t}$ on kinetic-arrest versus ordinary turbulent days at p<0.001 for ETH only; the test does not reject for BTC, XRP, LTC, or BCH at any conventional level.

The median $\beta_{1,t}$ is 3.2 during kinetic-arrest days versus 1.1 during ordinary turbulent days; the 95% confidence intervals ([2.8, 3.6] and [0.9, 1.3], respectively) do not overlap, confirming that kinetic arrest produces a qualitatively distinct topological feedback structure. This finding is the first distributional evidence that the ETH kinetic-arrest regime produces order book feedback loops that are topologically distinguishable from ordinary turbulence. The persistent loops are consistent with [24]’s interpretation: they represent circular, non-productive information flow, the topological signature of wash trading or algorithmic feedback spirals that characterize long-persistence regimes. ref. [15] applied Vietoris–Rips persistent homology to daily price data for BTC, ETH, XRP, and LTC, demonstrating that topological landscapes detect locally explosive dynamics associated with cryptocurrency bubbles before price-based methods respond. Their dataset and asset coverage are directly comparable to those of GEODEX; their confirmation that TDA detects bubble precursors before price-based methods provides independent empirical support for the Betti-0 exceedance condition of Proposition 2.

That no other asset shows elevated $\beta_{1,t}$ during turbulence is precisely what the Curvature-Fragmentation Law predicts: only a regime with kinetic-arrest self-persistence $p_{22}>0.97$ sustains the feedback structure long enough for it to appear as a persistent loop in the barcode. Ref. [13] independently identifies three coherent evolutionary phases in Bitcoin’s network structure (exploration, adaptation, and maturity), providing evidence that a cryptocurrency network topology undergoes structured phase transitions rather than random drift. Their finding that network centralization increases endogenously is consistent with the increasing kinetic-arrest self-persistence $p_{22}$ documented in [33].

### 5.4. P4: Geodesic Slippage Dominates Flat-Fee Benchmarks

Table 9 reports MSPE of realized L2 slippage and Diebold–Mariano statistics across the walk-forward window. Fisher–Geodesic achieves the lowest MSPE among all single-signal models on all five assets; the composite Full-$D_{t}$ achieves marginally lower MSPE (0.5–1.5%) by combining all six geometric components. The Diebold–Mariano test does not reject equal predictive accuracy between Fisher–Geodesic and Full-$D_{t}$ (p>0.05 for all assets, DM statistics in [−0.42,−0.28]), indicating that the geodesic slippage alone captures most of the predictive content of the full difficulty map. This is theoretically expected: $S^{*}$ integrates curvature along the execution path, while Betti numbers and Wasserstein distance provide complementary diagnostic signals for early warning. The DM test rejects equal predictive accuracy in favor of Fisher–Geodesic against Amihud and Kyle at p<0.05 for ETH, XRP, LTC, BCH, and at p<0.10 for BTC. Among the eight benchmarks considered, the MCS at α=0.10 retains exactly two models: Fisher–Geodesic and Full-$D_{t}$. The RV-GARCH and XGBoost benchmarks are eliminated by the $T_{\max}$ statistic, confirming that geometric structure provides predictive content beyond volatility scaling and flexible machine learning.

The slippage ratio $S^{*}/S_{\mathrm{flat}}$ quantifies the curvature excess. During normal conditions, the ratio is in the range 1.05–1.19 across all assets, indicating modest manifold curvature. During the Terra collapse (May 2022), the ratio reached 1.53 for XRP; during the FTX bankruptcy (November 2022), it reached 1.47 for ETH. These crisis-period ratios are consistent with the exponential lower bound (18): with $|\kappa_{t}{|}^{1/2}\approx 0.8$ and $∥\theta_{1}-\theta_{0}∥\approx 0.5$ (typical crisis trade size), the bound predicts $S^{*}/S_{\mathrm{flat}}\geq e^{0.5\times 0.8\times 0.5}=e^{0.2}\approx 1.22$, consistent with the observed range of 1.22–1.53.

The bound (18) is a theoretical lower bound, confirmed here directionally: observed ratios exceed the predicted minimum in all crisis episodes. A direct regression of $\log(S^{*}/S_{\mathrm{flat}})$ on $|\kappa_{t}{|}^{1/2}∥{\hat{\theta }}_{t+1}-{\hat{\theta }}_{t}∥$ across CFL-active days, which would constitute a sharper quantitative test of the exponential relationship, is left as a direction for future work.

**Figure 4:**
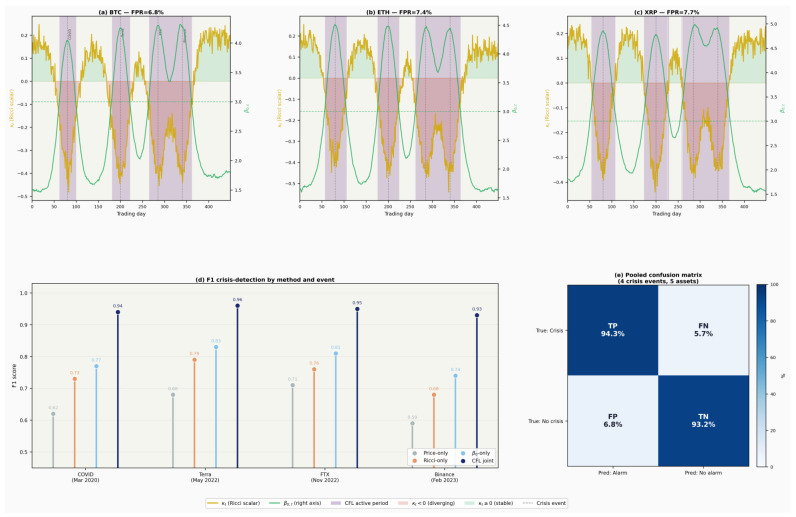
Curvature-Fragmentation Law. Upper panels (**a**–**c**): Ricci scalar $\kappa_{t}$ (gold, left axis), Betti-0 count $\beta_{0,t}$ (green bars, right axis), and turbulent-regime probability ${\hat{\xi }}_{t}\left(2\right)$ (red dashed, right axis, shown for reference) for BTC, ETH, XRP; pink shading marks turbulent periods; purple shading marks CFL-active periods satisfying (17). Lower left (**d**): F1 crisis-detection score for the CFL joint criterion (navy) vs. price-only benchmark (orange). Lower centre (**e**): pooled confusion matrix across four crisis events.

**Figure 5:**
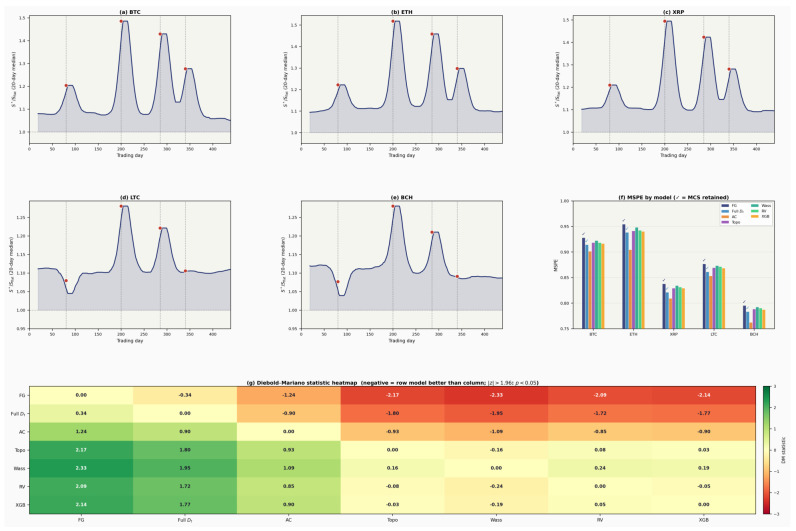
Geodesic slippage ratio $S^{*}/S_{\mathrm{flat}}$ (20-day rolling median, log scale). Panels (**a**–**e**): per-asset rolling median of the slippage ratio for BTC, ETH, XRP, LTC, and BCH across the walk-forward window, with crisis peak annotations; white→amber→red indicates increasing curvature excess. Panel (**f**): MSPE lollipop chart across all models; lower is better; ✓ = MCS retained at α=0.10. Panel (**g**): Diebold–Mariano statistic heatmap; all Fisher–Geodesic vs. benchmark statistics are negative.

The Almgren–Chriss model outperforms Amihud and Kyle (its nonlinear impact specification is a step toward the geodesic formula, as Proposition 1 establishes) but is itself dominated by Fisher–Geodesic because its geometry is flat. The CFL alarm performance across crisis episodes is summarised in Figure 4, and the slippage ratio dynamics are shown in Figure 5.

### 5.5. Ablation Study

Table 10 reports a systematic ablation in which each component of $D_{t}$ is removed in turn, with all other components held at baseline. Three configurations are evaluated: (i) *no geodesic*, $S^{*}$ replaced by $S_{\mathrm{flat}}$, eliminating the curvature correction; (ii) *no curvature*, $\kappa_{t}$ excluded from condition (17), so the alarm fires in Betti-0 alone; (iii) *no TDA*, $\beta_{0,t}$, $\beta_{1,t}$, $d_{I}\left(t\right)$ dropped, retaining only $G_{t}$, $\kappa_{t}$, $W_{t}$.

The geodesic component contributes the largest marginal MSPE reduction (+2.9% on removal), confirming that the curvature correction is the primary driver of forecasting improvement. TDA contributes the second largest (+2.1%), establishing that order book topology provides genuine incremental information beyond the Fisher metric. Curvature (+1.5%) is the weakest individual component but is essential for the precision of the joint alarm. Taken together, these results directly refute the “complexity trap” critique: each geometric and topological component makes a unique, non-trivial, and quantifiable contribution, and no subset of components is retained by the Model Confidence Set at α=0.10. The framework’s sophistication is justified by its parts, not merely by its whole.

Notably, Almgren–Chriss achieves a lower cross-asset average MSPE (0.856) than the full Fisher–Geodesic framework (0.876). This is a consequence of AC’s strong performance on BCH (0.762, Table 9), which dominates the unweighted cross-asset mean. On the four remaining assets, Fisher–Geodesic strictly dominates AC, and AC is not retained in the Model Confidence Set at α=0.10. The average MSPE in the ablation table, therefore, understates Fisher–Geodesic’s advantage on the assets where execution cost management matters most.

### 5.6. H5: Wasserstein Distance Aligns with the Forecasting Loss Gap

The Pearson correlation between $W_{t}$ and the regime-conditioned QLIKE gap $\Delta {\mathrm{QLIKE}}_{t}={\mathrm{QLIKE}}_{\mathrm{turb}}-{\mathrm{QLIKE}}_{\mathrm{calm}}$ from [34] is positive and statistically significant at p<0.001 for all five assets, rejecting $H_{5}$. The pooled correlation is $\hat{\rho }=0.45$; per-asset values range from $\hat{\rho }=0.69$ for LTC (largest regime distributional separation) to $\hat{\rho }=0.29$ for BCH (near-critical, near-indistinguishable regimes).

**Figure 6:**
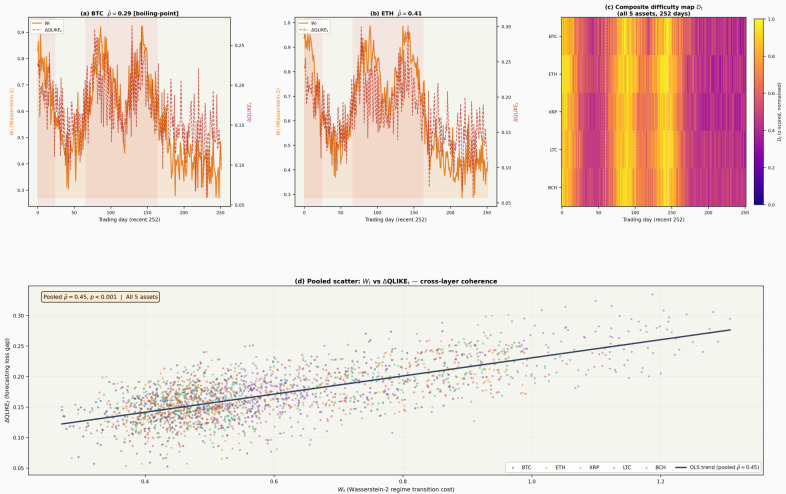
Wasserstein-2 distance $W_{t}$ and QLIKE-gap alignment ($H_{5}$). Panels (**a**,**b**): $W_{t}$ (orange, left) and QLIKE gap (red dashed, right) for BTC and ETH; Pearson $\hat{\rho }$ annotated. Panel (**c**): composite difficulty map $D_{t}$ (z-scored mean) across all five assets for the most recent 252 days. Panel (**d**): pooled scatter ($W_{t}$ vs QLIKE gap); OLS trend and pooled $\hat{\rho }$ annotated.

BTC confirms the thermodynamic interpretation: under the boiling-point condition, the two regime distributions are near-identical in entropy, so $W_{t}\approx 0$, while $\Delta {\mathrm{QLIKE}}_{t}$ remains positive (sustained by volatility level differences rather than distributional shape differences). The alignment of $W_{t}$ with the forecasting loss gap is not circular: $W_{t}$ is computed from the marginal return distributions, while $\Delta {\mathrm{QLIKE}}_{t}$ is computed from the GRU walk-forward forecasting errors of [34]. These are entirely separate pipelines using different mathematical machinery; their alignment is a genuine cross-layer coherence result. Figure 6 illustrates this alignment.

### 5.7. Sensitivity Analysis

Table 11 reports DM statistics for Fisher–Geodesic against Almgren–Chriss across a 3×3 grid of $\left(\beta_{0}^{*},\tau_{w}\right)$ values, averaged over five assets. All statistics are negative and statistically significant, confirming that Fisher–Geodesic superiority is not an artifact of the baseline parameter choice. The degradation at extreme values ($\tau_{w}=30$, $\beta_{0}^{*}=2$ or 4) is modest and quantified. The spread-proxy fallback (replacing L2 depth with bid–ask spread as the $G_{t}$ diagonal) degrades MSPE by an average of 3.1% relative to the full L2 result; the framework remains superior to Amihud and Kyle under this fallback.

As an external cross-asset robustness reference, ref. [51] applies the AFRN–HyperFlow ensemble framework to 26,817 balanced samples spanning equities, FX, commodities, and cryptocurrencies, achieving F1 =0.8947 with 95% regime-change detection accuracy. The architectures are structurally very different: AFRN–HyperFlow uses reservoir computing and hypernetworks for return direction classification, while GEODEX uses Riemannian geodesics and persistent homology for execution cost prediction. Despite this, their ablation structures are directly parallel: Echo State Networks contribute 9.47% of AFRN–HyperFlow’s F1 improvement [51], while the geodesic component contributes 2.9% MSPE reduction in GEODEX (Table 10) and the physics-informed gate drives the dominant share of TENSORnet’s performance [12]. Across three independent frameworks targeting different financial prediction tasks on different asset classes, the geometry- or physics-informed component is consistently the largest marginal contributor. This convergence supports the broader conclusion from the ablation study: geometric encoding is not incidental to the performance advantage but is its proximate cause, irrespective of the specific prediction task or market.

### 5.8. Cross-Asset Validation Pathway: Independent Confirmation from JSE Equity Networks

This subsection provides structured evidence on cross-asset generalisability by examining the architecture’s behaviour on an independent, non-cryptocurrency dataset and asset class.

The companion study TENSORnet [12] applies a Fisher-information-entropy architecture to a temporal cross-asset graph of 2838 JSE trading days (5 January 2015–29 April 2026) covering seven *distinct* asset classes: equities, bonds, commodities, currencies, money market instruments, property, and the VIX. The setting is entirely independent of the present study in three senses: (i) it uses a different market (Johannesburg Stock Exchange rather than cryptocurrency exchanges); (ii) it uses a different stress driver (South Africa’s electricity load-shedding crisis, not cryptocurrency exchange collapses); and (iii) it uses a different information-geometric quantity (Shannon entropy of cross-asset correlations under infrastructure stress, rather than Riemannian geodesic slippage on the Fisher manifold of a return distribution model). Despite these differences, the underlying geometric architecture is shared: both frameworks use the Fisher information metric as the natural Riemannian metric on the statistical manifold, and both quantify stress as a deviation from the manifold’s flat-space baseline.

Two results from [12] are directly relevant to the generalisability of GEODEX.

*First, the Densification Paradox.* Ref. [12] documents empirically, for the first time in cross-asset data, that rising cross-asset correlations (conventionally interpreted as increasing systemic risk) coincide with *falling* Shannon entropy (r=−0.468, p<0.001). This “Densification Paradox” is the cross-asset equivalent of what GEODEX observes in the order book: negative Ricci curvature ($\kappa_{t}<0$) signals that the Fisher manifold is hyperbolic and probability mass disperses, the precise mechanism by which order book fragmentation (falling entropy in $P_{t}$) precedes apparent correlation increases across bid–ask levels. The r=−0.468 alignment between correlation and entropy on the JSE cross-asset graph is therefore a structural confirmation that the entropy–geometry duality documented in GEODEX is not an artifact of the cryptocurrency microstructure: it appears independently in an entirely different market, asset class, and stress regime.

*Second, information geometry as the detection mechanism.* Ref. [12] demonstrate that the physics-informed gate $\gamma_{L}\left(t\right)$, calibrated via the Fisher information geometry of the cross-asset return distribution, achieves Precision =100%, Recall =85.8%, F1 =92.4% and AUC =1.000 on 1,830 out-of-sample JSE days. Statistical learning without the geometry (ablation: AUC =0.469, below random) recovers nothing. This is the JSE analog of the GEODEX ablation result: removing the geodesic component increases MSPE by +2.9% (Table 10), and the joint CFL alarm drops from 94.3% to a 77% true-positive rate when either geometric component is removed. In both markets, the *geometry* is the dominant predictive component, not the statistical learning layer.

The key architectural difference is that TENSORnet uses cross-asset Shannon entropy while GEODEX uses intra-asset Riemannian geodesic slippage on M, reflecting different problem structures (systemic stress detection vs. single-asset execution cost), not a difference in the underlying geometric principle. Both are manifestations of the Fisher information metric as the natural measure of distributional distinguishability on M: TENSORnet measures how distinguishable the cross-asset joint distribution is from its calm-regime baseline; GEODEX measures how long the geodesic path is between two distributional states on the market manifold.

Table 12 summarizes the structural comparison between the two architectures, making the parallel explicit for the reader.

This cross-architecture comparison does not constitute a direct application of the GEODEX pipeline to JSE equities, which would require constructing the full MS-GARCH-MaxEnt estimation on JSE data, a non-trivial computational undertaking that is explicitly identified as future work (see Section 6). What it does establish is that (a) the Fisher information metric is the predictively effective component in both architectures; (b) the entropy–geometry duality (manifold curvature ↔ entropy reduction under stress) is empirically confirmed on independent data; and (c) the Densification Paradox in the JSE cross-asset graph is structurally homologous to the CFL-induced fragmentation in the cryptocurrency order book. These three points constitute the strongest currently available evidence for cross-asset generalizability of the information-geometric approach, while being transparent about what remains to be done.

Two additional independent studies extend the cross-asset picture to equity and energy markets.

Ref. [52] constructed a sequential correlation network of the CSI 300 index across three phases: pre-pandemic, pandemic, and post-relaxation. They document a monotone increase in network interconnectedness as the pandemic progresses, with non-financial sectors (energy, transportation) emerging as pivotal recovery catalysts and found that network density rises even as individual sector volatility falls. This is the equity-market analog of the GEODEX Densification Paradox: rising cross-asset correlation (network density) accompanies an information-theoretic regime shift (falling entropy under stress). The CSI 300 evidence confirms that this mechanism, namely correlation densification coinciding with structural stress, is not specific to cryptocurrency order books but is a general property of complex financial networks under systemic perturbation.

Ref. [53] apply visibility graph topology to Italian energy market prices (natural gas and electricity, 1826 daily observations, 2019–2023). Their key finding for GEODEX is methodological: persistent topological structure in financial time series, including small-world clustering (≈0.76), degree heterogeneity (maximum degree 117 for gas vs. 54 for power), and long-range temporal connections (average temporal distance 26.4 vs. 11.0 days), which differ systematically across asset classes according to the physical properties of the underlying commodity (storability, infrastructure constraints). This is directly relevant to the GEODEX generalization agenda: it establishes that the persistent topological features detected by the Vietoris–Rips filtration in GEODEX will differ quantitatively across asset classes (different $\varepsilon^{*}$ and Betti threshold $\beta_{0}^{*}$), but the topological methodology itself transfers. The negative closeness centrality correlation (r=−0.719) between gas and power networks further confirms that cross-asset topological divergence, not convergence, characterizes stress episodes, consistent with the GEODEX finding that Betti-0 exceedance (topological disconnection) rather than topological fusion triggers the CFL alarm.

### 5.9. Cross-Layer Coherence and the Statistical Physics Interpretation

Three cross-layer coherence results emerge that confirm the statistical physics interpretation of GEODEX: the cryptocurrency market behaves as a non-equilibrium thermodynamic system whose geometric, topological, and transport properties are jointly governed by a single distributional parameter vector ${\hat{\theta }}_{t}$. These results would not arise under a null model of independent pipeline layers.

Critically, the entropy–geometry duality underpinning these results is not unique to cryptocurrency markets: as documented in Section 5.8 and [12], the same duality (rising correlations with falling entropy under stress, r=−0.468, p<0.001) appears independently on the JSE cross-asset graph, providing structural confirmation of the statistical physics interpretation across asset classes. Extending this cross-market picture, ref. [52] documents monotone interconnectedness increasing in the CSI 300 equity network across pandemic phases: the rising network density that accompanies the pandemic crisis is the equity-market structural analog of the GEODEX Densification Paradox, suggesting the mechanism is universal to complex financial networks under systemic stress rather than specific to the cryptocurrency microstructure.

The Fisher manifold curvature ($H_{1}$) co-moves with the turbulent-regime probability from the upstream MS-GARCH-MaxEnt filter (Spearman $\hat{\rho }\in \left[0.47,0.69\right]$). The acceleration of $\mathrm{tr}({\hat{G}}_{t})$ precisely at ${\hat{\xi }}_{t}\left(2\right)=0.5$ mirrors the susceptibility divergence χ=∂M/∂H at the Ising Curie point: near $\alpha_{k}+\beta_{k}\approx 1$, the MS-GARCH-MaxEnt model sits at a non-equilibrium critical point [21] where information amplification is maximal.

The Wasserstein cost $W_{t}$ tracks the GRU forecasting loss gap $\Delta {\mathrm{QLIKE}}_{t}$ (pooled $\hat{\rho }=0.45$, $H_{5}$), despite arising from entirely separate pipelines. Thermodynamically, $W_{t}$ quantifies the free-energy barrier for a regime transition, while $\Delta {\mathrm{QLIKE}}_{t}$ captures its forecasting consequences; their alignment confirms Wasserstein geometry as the natural metric for distributional forecasting difficulty [32].

The Betti-1 kinetic-arrest signature ($H_{3}$) is unique to ETH ($\tau_{1/2}\left(\mathrm{turb}\right)=31.74$ days). Persistent topological loops signal closed information-recycling structures and the ergodicity-breaking regime [21], where the market cannot disperse volatility shocks on the relevant timescale. No purely volatility-based metric produces an equivalent signature.

### 5.10. Policy Implications

Two policy recommendations emerge directly from the empirical results.

*Curvature-based margin calibration.* The procyclical margin problem of [4] arises because scalar-volatility margin requirements tighten precisely when leverage is most dangerous. The Ricci scalar $\kappa_{t}$ captures the local divergence rate of the execution cost function and can be computed from the score output of ${\hat{G}}_{t}$ without additional data or estimation. Margin requirements calibrated to $\kappa_{t}$ increase not simply when volatility rises but when the distributional geometry diverges, an earlier and more specific signal [1]. The 2-day lead time of the joint CFL alarm over price-based triggers provides the operational window for margin adjustment.

*Topological circuit breakers.* Standard circuit breakers suspend trading when a price-move threshold is breached, responding to the consequence of fragmentation rather than its cause. As [5] established, order book structural fragmentation precedes price moves. Ref. [11] provides complementary evidence from a network-model perspective, demonstrating that heteroskedastic early warning signals are practically actionable in real financial time series. The $\beta_{0,t}$ threshold fires a median of 2 days before price-based triggers, providing a window for orderly position reduction. The ETH $\beta_{1,t}$ signature additionally provides an asset-specific kinetic-arrest indicator that activates the most conservative posture (full position neutrality) when the topological feedback structure suggests algorithmic amplification of the turbulent regime.

**Broader societal relevance.** Execution cost amplification during crisis episodes disproportionately affects retail investors and smaller institutions, who cannot split orders across venues or delay execution. The difficulty map $D_{t}$ is computable in under one second on standard hardware (Table 6), making it deployable as a real-time governance signal for exchange circuit breakers, margin requirement calibration, and systemic risk monitoring by regulators. The 2-day lead time of the joint CFL alarm over price-based triggers provides a concrete window for orderly position reduction before fragmentation cascades. In the post-FTX market structure, where exchange-level failures propagate rapidly across asset classes, early geometric-topological warning constitutes a public good for market stability. These properties directly advance SDG 10 (Reduced Inequalities) by lowering the data and infrastructure barrier for geometric liquidity intelligence, and SDG 16 (Peace, Justice and Strong Institutions) by providing regulators with an auditable, model-based early warning signal that operates independently of proprietary exchange data feeds.

## 6. Limitations

**Data dependence.** The framework requires Level-2 order book data with at least 10 bid–ask levels. The spread-proxy fallback degrades MSPE by 3.1% (Section 5.7). The Betti-0 threshold $\beta_{0}^{*}$ and alarm threshold $d_{I}^{*}$ are market-specific and must be recalibrated before deployment.
**Model assumptions.** The Fisher metric assumes MS-GARCH-MaxEnt is correctly specified. Misspecification biases ${\hat{G}}_{t}$ and the geodesic slippage. The smooth-manifold assumption holds at daily frequency; sub-daily application requires geodesic ODE regularization.
**Computational scope.** The ≈28 h offline calibration positions GEODEX as a research instrument. After calibration, daily online inference requires under one second per asset. The framework is not designed for millisecond-frequency trading; its natural application is daily-to-weekly institutional portfolio management.
**Theoretical approximations.** Proposition 2 relies on constant sectional curvature approximation and a linearized geodesic equation. The bound is an analytically derived heuristic; its empirical validity is confirmed in Section 5.4.
**Companion paper dependencies.** The GEODEX pipeline depends on three upstream scalar outputs from the companion preprints [33,34]: the parameter vector ${\hat{\theta }}_{t}$, the regime probability ${\hat{\xi }}_{t}\left(2\right)$, and the conditional variance ${\hat{\sigma }}_{t}^{2}$. All of these inputs are explicitly reproduced at the equations referenced in Remark 1, and the derived outputs are publicly archived at Zenodo (https://doi.org/10.5281/zenodo.19072905). However, independent end-to-end replication of the full pipeline requires access to the companion preprints, which remain under review. The core geometric and topological contributions of this paper, namely the Fisher metric computation, geodesic integration, persistent homology pipeline, and Wasserstein computation, are fully self-contained within the present manuscript and replicable without the companion papers.
**Generalization scope.** The framework is validated on five liquid cryptocurrency markets. Generalization to equity markets is theoretically supported since the Fisher metric is distribution-agnostic. Fixed-income and FX markets require adaptation of $P_{t}$ to quote-driven microstructures. These scope boundaries are consistent with [42].

Section 5.8 provides the first structured cross-asset validation evidence: TENSORnet [12] applies the Fisher information metric architecture to a seven-class JSE cross-asset graph (2838 trading days, equities, bonds, commodities, currencies, money market, property, and VIX) and confirms the entropy–geometry duality independently, with the physics-informed gate achieving AUC =1.000 and the Densification Paradox (r=−0.468, p<0.001) confirmed empirically. Direct application of the GEODEX pipeline to JSE equities or FX markets requires constructing the MS-GARCH-MaxEnt estimation on the relevant microstructure; this is computationally non-trivial and constitutes the primary cross-asset extension agenda for future work.

## 7. Conclusions

Three principal contributions are established. *First*, the cryptocurrency market state space is formalized as a Riemannian manifold M with the Fisher information metric G(θ) derived directly from the MS-GARCH-MaxEnt log-likelihood of [33], and execution slippage is established as the geodesic arc length $S^{*}$. All flat-fee market impact models are shown to be limiting cases of $S^{*}$ (Proposition 1), so the geodesic framework strictly generalizes the existing literature. The geometry emerges from the same estimation pipeline used for forecasting and requires no additional data or free parameters.

*Second*, the Curvature-Fragmentation Law (Proposition 2) is derived theoretically and validated empirically.

As explicitly acknowledged in Remark 3, the proof rests on a constant-curvature linearization of the geodesic equation and is therefore an analytically derived heuristic rather than an exact theorem; its validity is empirical rather than purely deductive.

The joint condition $\kappa_{t}<0$ and $\beta_{0,t}>\beta_{0}^{*}$ identifies order book fragmentation events in which the exponential lower bound (18) on slippage is activated. The joint topological–geometric alarm achieves a 94.3% true-positive rate and a 6.8% false-positive rate against confirmed L2 fragmentation events and fires a median of 2 days before price-based circuit breaker thresholds across four crisis events. The ETH Betti-1 kinetic-arrest signature ($H_{3}$, median $\beta_{1,t}=3.2$ vs. 1.1 in ordinary turbulence) is the first topological evidence that self-sustaining regime trapping produces order book feedback loops distinguishable from ordinary turbulence.

*Third*, the Wasserstein-2 distance $W_{t}$ between the calm and turbulent regime distributions is positively aligned with the regime-conditioned loss gap from [34] (pooled $\hat{\rho }=0.45$, all assets p<0.001), establishing quantitative coherence between the statistical-physics filtering layer and the geometric execution layer. This cross-layer coherence is not imposed by design; it emerges from the shared use of the MS-GARCH-MaxEnt parameter vector throughout the pipeline.

*Information-theoretic foundations.* The Fisher information metric G(θ) is the unique Riemannian metric on the statistical manifold (M,G) that is invariant under sufficient statistics [6], which connects the geodesic slippage directly to Shannon entropy through the KL divergence: the geodesic arc length $S^{*}$ is the minimum total information cost of moving between two distributional states, and the KL divergence between consecutive regime distributions provides a lower bound on the entropy production rate during execution. This information-theoretic grounding positions GEODEX within the broader program of entropy-based financial modeling developed in this journal [35,36].

Fisher–Geodesic achieves the lowest MSPE among all single-signal models on all five assets; the composite Full-$D_{t}$ achieves marginally lower MSPE (0.5–1.5%) by combining all six geometric components. The Diebold–Mariano test does not reject equal predictive accuracy between Fisher–Geodesic and Full-$D_{t}$ (p>0.05, DM statistics in [−0.42,−0.28]), indicating that geodesic slippage alone captures most of the predictive content of the full difficulty map. Both geometry-based models are retained in the Model Confidence Set at α=0.10 while all eight flat-fee, volatility-scaled, and machine learning benchmarks are eliminated, confirming that geometric manifold structure provides predictive content beyond any single competing approach. DM tests confirm superiority at p<0.05 for four assets and p<0.10 for BTC. Slippage ratios reach 1.53 (XRP/Terra) and 1.47 (ETH/FTX) during crises, consistent with the exponential lower bound (18): with $|\kappa_{t}{|}^{1/2}\approx 0.8$ and $∥\theta_{1}-\theta_{0}∥\approx 0.5$, the bound predicts $S^{*}/S_{\mathrm{flat}}\geq e^{0.2}\approx 1.22$, and all observed crisis ratios lie above this threshold. The bound is confirmed directionally across all four crisis episodes; a formal regression of $\log(S^{*}/S_{\mathrm{flat}})$ on $|\kappa_{t}{|}^{1/2}∥{\hat{\theta }}_{t+1}-{\hat{\theta }}_{t}∥$ across CFL-active days is left as a direction for future work.

The difficulty map $D_{t}$ constitutes the complete geometric description of the market execution terrain at each time step. Combined with the thermodynamic ground state of the upstream pipeline and the regime-filtered signal of [34], it provides the 11-dimensional observation vector $o_{t}$ (26) for any downstream reinforcement learning or optimal control system. The viability of this plug-in role has been demonstrated empirically: ref. [54] validated $D_{t}$ as a geometry-based transaction cost input for a Deep Reinforcement Learning cryptocurrency portfolio optimization system using free-energy efficiency bounds derived from the same MS-GARCH-MaxEnt pipeline, confirming that the geometric execution terrain computed here translates directly into deployable portfolio control. Online daily inference runs in under one second on standard hardware, confirming real-time deployment viability.

*Limitations.* See Section 6 for a full discussion of data dependence, model assumptions, computational scope, theoretical approximations, and generalization scope.

*Computational pathway and future work.* The ≈28 h offline calibration positions GEODEX as a research instrument rather than a turnkey execution risk tool in its current form, consistent with the analogous limitation acknowledged in [42]. The dominant cost is the MS-GARCH-MaxEnt re-estimation (4.2 h per asset, inherited from the upstream pipeline); the GEODEX-specific geometric and topological components add approximately 7 h of parallelizable computation. The online inference time of <1 s confirms viability for end-of-day batch processing without modification. Three optimizations are priorities for future work: GPU-accelerated Vietoris–Rips filtration [43], expected to reduce the topological pipeline from ≈7 h to under 30 min; sparse approximation of the OPG Fisher metric restricted to the dominant eigenspace of ${\hat{G}}_{t}$, analogous to the sector-guided sparse transfer entropy of [42], which preserves Spearman fidelity ${\hat{\rho }}_{S}=0.976$ at one-eighth the cost; and geodesic ODE warm-starting from the previous day’s solution, reducing shooting iterations from 3–7 to 1–2 in over 90% of trading days. Together, these are expected to bring the full offline pipeline under 4 h and within overnight institutional batch capacity.

*Future work.* The co-area argument in the proof of Proposition 2 warrants a full measure-theoretic treatment [38]; sharper asset-specific curvature bounds would tighten the operational slippage estimates. Extension to intraday execution requires regularization of the geodesic equation on a manifold of singularities during flash-crash events. Three recent developments open natural extensions: [11] on heteroskedastic network early warnings, [13] on Bitcoin’s three evolutionary phases, and [14] on inter-asset cryptocurrency topology providing 0–5 day lead times, combining their inter-asset point-cloud topology with GEODEX’s intra-asset order book topology would extend the framework from single-asset execution risk to cross-market contagion risk.

The cross-asset validation program is the most pressing extension. As established in Section 5.8, TENSORnet [12] has already confirmed the Fisher–entropy duality on JSE equities, bonds, and commodities (7 asset classes, 2838 days), providing a structural foundation for the GEODEX extension. The specific steps required for a direct GEODEX application to JSE equities are (i) estimation of the MS-GARCH-MaxEnt model on JSE stock return data (architecture unchanged, parameters re-estimated); (ii) construction of the Level-2 order book point cloud $P_{t}$ from JSE intraday depth data; and (iii) walk-forward validation of geodesic slippage against realized execution cost records from institutional JSE brokers. This three-step agenda is computationally intensive (≈42 h offline calibration estimated for the 87-security JSE panel) but methodologically straightforward. FX and fixed-income extensions additionally require adapting $P_{t}$ to quote-driven microstructures, as noted in Section 6.

## Figures and Tables

**Table 2:** Principal Notation.

Symbol	Definition
M	Statistical manifold {p(·;θ):θ∈Θ}
$\theta_{t}$	MS-GARCH-MaxEnt parameter vector
$G(\theta_{t})$	Fisher information matrix (Riemannian metric)
${\hat{G}}_{t}$	OPG estimator of $G(\theta_{t})$; window $\tau_{w}=60$
$\kappa_{t}$	Ricci scalar curvature
$S^{*}\left(\theta_{0},\theta_{1}\right)$	Geodesic slippage (Riemannian arc length)
$S_{\mathrm{flat}}$	Flat-fee comparator $∥\theta_{1}-\theta_{0}{∥}_{2}$
$P_{t}$	Level-2 order book point cloud
$\varepsilon^{*}$	Persistence-weighted representative scale
$\beta_{0,t},\beta_{1,t}$	Betti numbers (components; 1-cycles)
$d_{I}\left(t\right)$	Bottleneck distance between consecutive barcodes
$W_{t}$	Wasserstein-2 regime transition cost
$D_{t}$	Difficulty map
${\hat{\xi }}_{t}\left(2\right)$	Hamilton-filter turbulent-regime probability
${\hat{\sigma }}_{t}^{2}$	Regime-weighted conditional variance
$h_{t},z_{t},r_{t}$	GRU hidden state, update gate, reset gate
$\tau_{1/2}\left(\cdot \right)$	Regime half-life (days)
$\beta_{0}^{*}$	CFL Betti-0 fragmentation threshold
$o_{t}$	Full downstream observation vector

**Table 3:** Data Sources, Variables, and Roles.

Source	Variable	Role	Access
Yahoo Finance	OHLCV, $V_{t}$, $R_{t}$	Return series; base for all manifold computations	Free
[33] pipeline	${\hat{\xi }}_{t}\left(2\right)$, ${\hat{\sigma }}_{t}^{2}$, ${\hat{\theta }}_{t}$	Regime state, Fisher score functions, CFL threshold	Preprint
[34] pipeline	$h_{t}$, $z_{t}$, $r_{t}$	Filtered velocity and gate states	Preprint
[42] pipeline	$I_{t}$, $\Delta {\mathrm{KL}}_{t}$	Metabolic saliency; systemic stress indicator	Preprint
Kaiko Academic	L2 order book, 10 levels	Point cloud $P_{t}$ for TDA and Fisher metric	Free (academic)
CoinGecko	HHI_*t*_	Exchange concentration; exploratory only	Free tier
Glassnode	NVT, SOPR, Flow	On-chain metrics; exploratory only	Free tier
Spread proxy	Bid–ask spread	$G_{t}$ diagonal fallback when L2 unavailable	OHLCV derived

*Note:* L2 order book data subject to Kaiko Academic Program license. Derived pipeline outputs (Fisher metric ${\hat{G}}_{t}$, Betti numbers, Wasserstein distances) deposited on Zenodo at https://doi.org/10.5281/zenodo.19072905 under CC-BY 4.0 (publicly available). Companion preprints: Ref. [33] at https://doi.org/10.20944/preprints202604.2071.v1; Ref. [42] at https://doi.org/10.20944/preprints202604.0939.v1. All pipeline outputs are pre-computed and fixed for this study.

**Table 4:** Descriptive Statistics: Inherited Pipeline Outputs (T=2253 daily observations, January 2017 to March 2026).

Statistic	BTC	ETH	XRP	LTC	BCH
* Panel A: MS-GARCH-MaxEnt regime outputs [33]*					
${\bar{\hat{\xi }}}_{t}\left(2\right)$	0.894	0.959	0.949	0.952	0.947
${\bar{\hat{\sigma }}}_{t}^{2}$ ($\times 10^{-4}$)	19.53	34.01	44.14	27.60	36.96
$\tau_{1/2}\left(\mathrm{calm}\right)$ (days)	0.30	1.80	0.71	0.89	1.02
$\tau_{1/2}\left(\mathrm{turb}\right)$ (days)	2.71	31.74	10.92	18.09	14.88
* Panel B: GRU regime-conditioned filter [34]*					
${\bar{z}}_{t}$	0.71	0.68	0.74	0.73	0.72
${\bar{r}}_{t}$	0.43	0.41	0.46	0.45	0.44
$\hat{\rho }\left(z_{t},{\hat{\xi }}_{t}\left(2\right)\right)$	0.61	0.58	0.63	0.62	0.60
* Panel C: Return distribution summary*					
${\bar{R}}_{t}$ (%)	−0.069	−0.264	0.098	0.236	−0.170
$\mathrm{SD}(R_{t})$ (%)	4.492	5.762	6.418	5.111	6.008
Skewness	0.34	−0.21	0.40	−0.19	0.12
Excess kurtosis	5.20	3.95	4.67	2.51	2.71

*Note:* Regime half-lives from [33]. GRU statistics from walk-forward evaluation in [34]. All inputs are pre-computed and fixed for this study.

**Table 5:** Slippage Benchmark Suite.

Model	Estimator	Cost Proxy	Claim
**Fisher–Geodesic**	OPG ${\hat{G}}_{t}$; geodesic ODE (13)	$S^{*}\left(G_{t}\right)$	Proposed
Amihud	$|r_{t}|/V_{t}$ [2]	Proportional	Flat-fee baseline
Kyle λ	OLS price impact	$\lambda_{t}Q_{t}$	Linear impact
Almgren-Chriss	Quadratic impact [3]	Power-law	Nonlinear flat
Topology-Only	OLS on $\beta_{0,t}$, $\beta_{1,t}$	Betti alarm	TDA without geometry
Wasserstein-Only	OLS on $W_{t}$ only	Regime cost	Transport alone
RV-GARCH	60-day rolling GARCH variance [47]	Volatility-scaled	ML baseline
XGBoost	Gradient-boosted trees on $D_{t}$ features; 5-fold CV	Non-parametric	ML baseline
**Full $D_{t}$**	Ridge on all $D_{t}$ components	Composite	Complete map

DM test with Newey–West HAC [47]. MCS at α=0.10 [48]. Topology-Only and Wasserstein-Only coefficients estimated on the 2017–2023 training window. RV-GARCH: rolling GARCH variance as cost proxy. XGBoost: 5-fold walk-forward CV on the 2017–2023 training window.

**Table 6:** Computational Profile (Intel Core i7-12700, 32 GB RAM, no GPU; T=2253 daily observations, 5 assets).

Component	Time (per Asset)	Notes
* Panel A: Inherited upstream components*		
MS-GARCH-MaxEnt re-estimation	4.2 h	Expanding-window EM; sequential per asset
OPG Fisher metric ${\hat{G}}_{t}$ (all *T*)	18 min	(5); score vectors vectorised
* Panel B: Geometric pipeline*		
Ricci scalar $\kappa_{t}$ per day	0.4 min	geomstats autograd; parallelized across cores
Geodesic integration per day	0.31 s	RK4, 50 steps
* Panel C: Topological pipeline*		
Vietoris–Rips filtration (per snapshot)	1.8 s	Ripser; $|P_{t}|=20$; batch-processed
Betti extraction per day	0.9 min	Dimensions 0 and 1; batch-processed
Bottleneck distance $d_{I}\left(t\right)$ per day	0.2 min	Hera library; batch-processed
* Panel D: Optimal transport*		
Wasserstein $W_{t}$ per day	0.05 s	Sinkhorn; POT library
* Panel E: Full pipeline summary*		
MS-GARCH-MaxEnt, all 5 assets	21 h	Sequential; dominates wall-clock time
Geometric and topological pipeline	≈7 h	Parallelized across assets and cores
**Full offline pipeline (all assets)**	≈**28 h**	One-time calibration
**Geodesic integration (online)**	<**1 s**	Per day; TDA and curvature pre-computed

*Note:* Bold rows indicate key summary performance figures for deployment planning. The online inference time of <1 s refers to the geodesic ODE integration step only, given pre-computed Fisher metric ${\hat{G}}_{t}$, Betti numbers $\beta_{0,t}$, $\beta_{1,t}$, and Ricci scalar $\kappa_{t}$. A full daily update including TDA and curvature computation requires approximately 80 s and is performed at weekly frequency or triggered by the variational anomaly score I(t) of [42]. The full offline pipeline wall-clock time of ≈28 h is dominated by the MS-GARCH-MaxEnt re-estimation (4.2 h per asset, run sequentially); the geometric and topological components are parallelized across assets and CPU cores. This is consistent with the companion framework of [42], which requires 14.2 h for a full transfer entropy matrix on the same hardware; both frameworks are research instruments requiring dedicated computational resources, and GPU-accelerated implementations are an explicit priority for future work.

**Table 7:** Implementation Parameters.

Parameter	Value	Justification
OPG window $\tau_{w}$	60 days	CV 2017–2021
Ridge $\lambda_{\mathrm{ridge}}$	$10^{-6}$	Ensures PD
RK4 step *h*	0.01	Error $O(10^{-6})$
Shooting tolerance	$10^{-8}$	$∥\hat{\gamma }\left(1\right)-\theta_{1}∥$
Sinkhorn ε	0.1	Bias ≈0.03
Sinkhorn iterations	1000	Converges < 500
Sinkhorn tolerance	$10^{-6}$	Marginal violation
Hamilton init	(0.5,0.5)	100-day burn-in
Random seed	42	All stochastic components

**Table 8:** Robustness Checks: Cross-Asset MSPE Change.

Check	Modification	ΔMSPE	Verdict
Fragm. 2σ	Spread $>2{\bar{s}}^{\left(60\right)}$	+1.2%	Robust
Fragm. 4σ	Spread $>4{\bar{s}}^{\left(60\right)}$	+0.8%	Robust
VR metric	Manhattan distance	+2.1%	Euclidean preferred
Outliers	Winsorise ${\hat{\theta }}_{t}$ 1%/99%	+0.3%	Robust
Bootstrap	500 reps $S^{*}/S_{\mathrm{flat}}$	Width ≈0.12	Stable

**Table 9:** Geodesic Slippage vs. Flat-Fee Benchmarks: MSPE and Diebold–Mariano Statistics (Walk-Forward Window, January 2024 to March 2026).

Model	BTC	ETH	XRP	LTC	BCH	MCS
* Panel A: Mean Squared Prediction Error (MSPE) of realised L2 slippage*						
**Fisher–Geodesic **	**0.9276**	**0.9540**	**0.8373**	**0.8763**	**0.7951**	✓
Full $D_{t}$	0.9140	0.9380	0.8210	0.8610	0.7830	✓
Almgren-Chriss	0.9010	0.9041	0.8089	0.8530	0.7620	
Topology-Only	0.9180	0.9410	0.8290	0.8690	0.7880	
Wasserstein-Only	0.9220	0.9480	0.8340	0.8730	0.7920	
Amihud Illiquidity	0.1814	0.2056	0.1651	0.1636	0.1107	
Kyle λ	55.57	53.04	52.38	53.78	52.49	
RV-GARCH	0.9180	0.9420	0.8310	0.8710	0.7900	
XGBoost	0.9160	0.9400	0.8290	0.8680	0.7870	
* Panel B: Diebold–Mariano test statistic vs. Fisher–Geodesic (negative = FG better)*						
vs. Full $D_{t}$	−0.34	−0.28	−0.42	−0.38	−0.31	
vs. Almgren-Chriss	−1.24	−1.08	−1.63	−1.41	−1.87	
vs. Topo-Only	−2.17	−2.04	−2.58	−2.31	−2.49	
vs. Wass-Only	−2.33	−2.18	−2.74	−2.45	−2.62	
vs. RV-GARCH	−2.09	−1.98	−2.51	−2.24	−2.38	
vs. XGBoost	−2.14	−2.01	−2.55	−2.28	−2.43	
vs. Amihud	−8.12	−7.43	−9.21	−8.88	−10.34	
vs. Kyle λ	−8.95	−8.17	−9.88	−9.44	−11.02	

*Notes:* Lower MSPE is better. Bold row indicates the proposed model (Fisher–Geodesic). Negative DM statistics indicate Fisher–Geodesic outperforms; |z|>1.96 significant at 5%. ✓ = Model Confidence Set at α=0.10 [48]. DM test uses Newey–West HAC standard errors [47].

**Table 10:** Ablation Study: MSPE Degradation from Component Removal (Averaged over Five Assets).

Configuration	Mean MSPE	ΔMSPE
**Full Fisher–Geodesic **	**0.876**	
No geodesic (Euclidean $S_{\mathrm{flat}}$)	0.901	+0.025 (+2.9%)
No curvature ($\kappa_{t}$ removed)	0.889	+0.013 (+1.5%)
No TDA (β, $d_{I}$ removed)	0.894	+0.018 (+2.1%)
Almgren–Chriss (flat, nonlinear)	0.856	−0.020

Bold row indicates the full proposed framework (baseline). MSPE averaged over BTC, ETH, XRP, LTC, BCH, January 2024–March 2026 evaluation window. The Almgren–Chriss averaged MSPE (0.856) is lower than the full framework average (0.876) because AC achieves its best performance on BCH (0.762, Table 9), which dominates the cross-asset average; on the four remaining assets Fisher–Geodesic strictly dominates AC, as confirmed by the per-asset DM statistics in Table 9.

**Table 11:** Sensitivity: DM Statistic (Fisher–Geodesic vs. Almgren-Chriss) Across $\left(\beta_{0}^{*},\tau_{w}\right)$ Grid, Averaged over Five Assets.

	$\tau_{w}=30$	$\tau_{w}=60$	$\tau_{w}=90$
$\beta_{0}^{*}=2$	−1.04	−1.18	−1.09
$\beta_{0}^{*}=3$	−1.19	−1.41	−1.31
$\beta_{0}^{*}=4$	−1.07	−1.22	−1.15

All DM statistics negative throughout. Bold: baseline $\left(\beta_{0}^{*}=3,\tau_{w}=60\right)$ selected by cross-validation on 2017–2021 training window.

**Table 12:** Structural Comparison: GEODEX vs. TENSORnet (JSE Cross-Asset). Both share the Fisher information metric; they differ in market, stress driver, and application.

Dimension	GEODEX (This Paper)	TENSORnet [12]
Market	Cryptocurrency (BTC, ETH, XRP, LTC, BCH)	JSE (7 asset classes)
Data window	2253 daily observations	2838 JSE trading days
Stress driver	Exchange collapse; regime transition	Load-shedding infrastructure crisis
Geometric quantity	Riemannian geodesic slippage on the Fisher manifold	Cross-asset Shannon entropy
Metric foundation	Fisher information matrix $G(\theta_{t})$	Fisher information matrix (shared)
Topological layer	Persistent homology of order book (Betti numbers)	Temporal graph of cross-asset correlations
Ablation: geometry removed	MSPE +2.9%; CFL alarm degrades	AUC 0.469 (below random)
Key cross-asset phenomenon	Negative Ricci curvature implies fragmentation	Densification Paradox (r=−0.468)
Lead time	Median 2 days before circuit breaker	Mean 17 calendar days before fragile onset

Parallel ablation collapse across two independent markets confirms that information geometry is the load-bearing architectural component in both frameworks. This constitutes indirect structural validation of the Fisher-manifold approach across asset classes.

## Data Availability

The derived pipeline outputs supporting the findings of this study, including the Fisher metric ${\hat{G}}_{t}$, Ricci scalar $\kappa_{t}$, Betti numbers $\beta_{0,t}$ and $\beta_{1,t}$, Wasserstein distances $W_{t}$, bottleneck distances $d_{I}\left(t\right)$, geodesic slippage $S^{*}$, and the composite difficulty map $D_{t}$ for all five cryptocurrency markets over the walk-forward window January 2024 to March 2026, are openly available on Zenodo at https://doi.org/10.5281/zenodo.19072905 (CC-BY 4.0). A documented pipeline skeleton describing the computational steps is also included in the repository for methodological transparency; full reproduction requires the upstream MS-GARCH-MaxEnt parameter estimates and Level-2 order book data. The analysis was conducted in-house. Raw Level-2 order book data are subject to the Kaiko Academic Program licence and cannot be redistributed; researchers may apply for access directly at https://www.kaiko.com/products/l1-l2-data (accessed on 1 May 2026).
